# Ovarian reserve in reproductive-aged patients with cancer before gonadotoxic treatment: a systematic review and meta-analysis

**DOI:** 10.1093/hropen/hoad024

**Published:** 2023-05-18

**Authors:** Meng Wu, Qingqing Zhu, Yibao Huang, Weicheng Tang, Jun Dai, Yican Guo, Jiaqiang Xiong, Jinjin Zhang, Su Zhou, Fangfang Fu, Mingfu Wu, Shixuan Wang

**Affiliations:** Department of Obstetrics and Gynecology, Tongji Hospital, Tongji Medical College, Huazhong University of Science and Technology, Wuhan, Hubei, China; Department of Obstetrics and Gynecology, Tongji Hospital, Tongji Medical College, Huazhong University of Science and Technology, Wuhan, Hubei, China; Department of Obstetrics and Gynecology, Tongji Hospital, Tongji Medical College, Huazhong University of Science and Technology, Wuhan, Hubei, China; Department of Obstetrics and Gynecology, Tongji Hospital, Tongji Medical College, Huazhong University of Science and Technology, Wuhan, Hubei, China; Department of Obstetrics and Gynecology, Tongji Hospital, Tongji Medical College, Huazhong University of Science and Technology, Wuhan, Hubei, China; Department of Obstetrics and Gynecology, Tongji Hospital, Tongji Medical College, Huazhong University of Science and Technology, Wuhan, Hubei, China; Department of Obstetrics and Gynecology, Zhongnan Hospital of Wuhan University, Wuhan, Hubei, China; Department of Obstetrics and Gynecology, Tongji Hospital, Tongji Medical College, Huazhong University of Science and Technology, Wuhan, Hubei, China; Department of Obstetrics and Gynecology, Tongji Hospital, Tongji Medical College, Huazhong University of Science and Technology, Wuhan, Hubei, China; Department of Obstetrics and Gynecology, Tongji Hospital, Tongji Medical College, Huazhong University of Science and Technology, Wuhan, Hubei, China; Department of Obstetrics and Gynecology, Tongji Hospital, Tongji Medical College, Huazhong University of Science and Technology, Wuhan, Hubei, China; Department of Obstetrics and Gynecology, Tongji Hospital, Tongji Medical College, Huazhong University of Science and Technology, Wuhan, Hubei, China

**Keywords:** ovarian reserve, ovarian function, AMH, inhibin B, AFC, FSH, cancer, fertility preservation, oncofertility, reproductive age

## Abstract

**STUDY QUESTION:**

Does cancer itself, before any gonadotoxic treatment, affect ovarian function in reproductive-aged patients?

**SUMMARY ANSWER:**

Our study revealed that women with cancer may have decreased ovarian reserve markers even before cancer therapy.

**WHAT IS KNOWN ALREADY:**

With the field ‘oncofertility’ improving rapidly, cancer therapy-mediated ovarian damage is well characterized. However, there is a controversy about whether cancer itself affects ovarian function before gonadotoxic treatment.

**STUDY DESIGN, SIZE, DURATION:**

We conducted a systematic meta-analysis investigating the association between cancer and ovarian function prior to gonadotoxic treatment. Titles or abstracts related to ovarian reserve (e.g. anti-Müllerian hormone (AMH), antral follicle count (AFC), or basal follicle-stimulating hormone (FSH)) combined with titles or abstracts related to the exposure (e.g. cancer*, oncolog*, or malignan*) were searched in PubMed, Embase, and Web of Science databases from inception to 1 February 2022.

**PARTICIPANTS/MATERIALS, SETTING, METHODS:**

We included cohort, case-control, and cross-sectional studies in English that examined ovarian reserve in reproductive-aged patients (18–45 years) with cancer compared to age-matched controls before cancer treatment. The quality of the included studies was assessed by ROBINS-I. Fixed or random effects were conducted to estimate standard or weighted mean difference (SMD or WMD, respectively) and CI. Heterogeneity was assessed by the *Q* test and *I*^2^ statistics, and publication bias was evaluated by Egger’s and Begg’s tests.

**MAIN RESULTS AND THE ROLE OF CHANCE:**

The review identified 17 eligible studies for inclusion. The results showed that cancer patients had lower serum AMH levels compared to healthy controls (SMD = −0.19, 95% CI = −0.34 to −0.03, *P *=* *0.001), especially women with hematological malignancies (SMD = −0.62, 95% CI = −0.99 to −0.24, *P *=* *0.001). The AFC was also decreased in patients with cancer (WMD = −0.93, 95% CI = −1.79 to −0.07, *P* = 0.033) compared to controls, while inhibin B and basal FSH levels showed no statistically significant differences.

**LIMITATIONS, REASONS FOR CAUTION:**

Serum AMH and basal FSH levels in this meta-analysis showed high heterogeneity, and the small number of studies contributing to most subgroup analyses limited the heterogeneity analysis. Moreover, the studies for specific cancer subtypes may be too small to draw conclusions; more studies are needed to investigate the possible impact of cancer type and stage on ovarian function.

**WIDER IMPLICATIONS OF THE FINDINGS:**

Our study confirmed the findings that cancer per se, especially hematological malignancies, negatively affects serum AMH level, and AFC values of reproductive-aged women. However, the lower AMH levels and AFC values may also be due to the changes in ovarian physiology under oncological conditions, rather than actual lower ovarian reserves. Based on the meta-analysis, clinicians should raise awareness about the possible need for personalized approaches for young women with cancer who are interested in pursuing fertility preservation strategies before anticancer treatments.

**STUDY FUNDING/COMPETING INTEREST(S):**

This work was financially supported by the National Natural Science Foundation of China (nos 81873824, 82001514, and 81902669) and the Applied Basic Research Program of Wuhan Municipal Bureau of Science and Technology (2019020701011436). The authors declare that they have no conflicts of interest.

**REGISTRATION NUMBER:**

PROSPERO (CRD42021235954).

WHAT DOES THIS MEAN FOR PATIENTS? Over the past decades, the incidence of cancer in young women has increased constantly. Cancer therapy-mediated damage to the ovary is well characterized, but whether cancer itself, prior to cytotoxic treatment, affects ovarian function is controversial. Ovarian reserve is generally defined as the quantity of oocytes remaining in the ovary, and markers of ovarian reserve include the hormone levels of anti-Müllerian hormone (AMH), inhibin B, and basal follicle-stimulating hormone (FSH) and the sonographically measured antral follicle count (AFC). Among these, AMH and AFC are the most favorable indicators of ovarian reserve. Based on the results of this meta-analysis, patients with cancer had significant decreased serum AMH levels and AFC values before cancer treatment, while basal FSH levels and inhibin B levels showed no difference between cancer patients and control groups. From these results, we suggest that cancer itself may have negative effects on ovarian reserve. These findings may provide valuable information to clinicians and patients for choosing effective fertility preservation measures before cancer treatment.

## Introduction

The incidence of cancer has increased over the past decades, and there is a rising trend in young women (Pilleron *et al.*, 2021). A well-known long-term adverse effect of cancer treatment in reproductive-aged female cancer survivors is gonadal dysfunction, which often results in premature ovarian insufficiency (POI) (Spears *et al.*, 2019; Xiong *et al.*, 2021). Currently, a growing body of clinical evidence suggests that cancer itself may have negative effects on reproductive function. A previous study reported that cancer, especially lymphoma, adversely affects the male reproductive system prior to the initiation of therapy (Fitoussi *et al.*, 2000). This relationship may be due to a direct cytotoxic effect of the cancer itself on testicular function or alterations of the immunological response (Rueffer *et al.*, 2001). However, whether cancer per se impairs ovarian reserve remains controversial.

Ovarian reserve is generally defined as the quantity of oocytes remaining in the ovary (Broer *et al.*, 2014). Markers of ovarian reserve include hormone levels (serum anti-Müllerian hormone (AMH), inhibin B, basal follicle-stimulating hormone (FSH)) and sonographically measured features (antral follicle count (AFC)) of the ovaries (Steiner *et al.*, 2017; Tal and Seifer, 2017; Younis *et al.*, 2020). Among them, AMH and AFC have demonstrated the most favorable analytical and performance characteristics as indicators of ovarian reserve (Iliodromiti *et al.*, 2015). Serum AMH levels remain consistent throughout the menstrual cycle, with no significant variability between the follicular and luteal phases, and levels are not affected by short-term oral contraception (OCP) (Broer *et al.*, 2014; Dewailly *et al.*, 2014). In contrast, AFCs vary across cycles and exhibit limited utility for studies in cancer patients (Iliodromiti *et al.*, 2015). However, both AMH and AFC show considerable variability in their clinical definitions and technical methods, which have not been standardized internationally (Practice Committee of the American Society for Reproductive Medicine, 2020). Researchers have recently focused on the effects of cancer on ovarian reserve. Some clinical studies have observed lower AMH levels in reproductive-aged women with cancer before treatment compared with unaffected women (Lutchman Singh *et al.*, 2007; Lie Fong *et al.*, 2008; Bala *et al.*, 2016; Naasan *et al.*, 2016; Paradisi *et al.*, 2016a,b; Decanter *et al.*, 2018; Porcu *et al.*, 2020). Similarly, Decanter et al. (2018) showed that AFC was significantly lower in cancer patients. In contrast, other studies have reached the opposite conclusion, having observed no effect of cancer on ovarian reserve (Paradisi *et al.*, 2016a,b; Goldrat *et al.*, 2019; Gunnala *et al.*, 2019; Hussein *et al.*, 2021). Therefore, no definite conclusion about ovarian reserve before gonadotoxic treatments in patients with cancer can be drawn.

To shed light on this issue, we performed a systematic review and meta-analysis to clarify the relationship between cancer and ovarian reserve before anticancer treatment. We aimed to analyze ovarian reserve function in patients with different types of cancer prior to treatment. To our knowledge, this is the first meta-analysis of case-controlled studies assessing ovarian reserve in patients with cancer and comparing the outcomes with those in age-matched infertile or healthy women. Our results may provide some guidance on fertility protection for patients even before cancer treatment.

## Materials and methods

We performed this systematic review and meta-analysis in accordance with the Preferred Reporting Items for Systematic Reviews and Meta-Analysis (PRISMA) guidelines. The protocol for this systematic review and meta-analysis was registered in PROSPERO (Registration ID Number CRD42021235954).

### Literature search

We performed a broad-range search strategy for eligible research articles in English in the PubMed, Embase and Web of Science databases from inception to 1 February 2022. Titles or abstracts related to ovarian reserve (e.g. AMH, AFC, or FSH) were combined with titles or abstracts related to exposure (e.g. cancer*, oncolog*, or malignan*). The full search strategy is shown in Supplementary Table S1. We also searched the reference lists of selected publications to retrieve additional studies that were not identified in the database search.

### Study selection

Cohort, case–control, and cross-sectional studies that examined ovarian reserve between patients with cancer and age-matched controls in the same setting, overall and/or by cancer type were included. The inclusion criteria were patients with mean age 18–45 years, no chemotherapy and/or radiotherapy, and no ovarian surgery, such as ovarian cystectomy or unilateral oophorectomy. Women taking OCP which may influence the ovarian reserve were excluded. Studies about women with a history of ovarian cancer, endometriosis, polycystic syndrome, or other ovarian diseases were excluded. Case series, case reports, reviews, meta-analyses, commentaries, letters, and animal studies were also excluded. Studies that compared ovarian reserve in women with cancer to a non-age-matched control group or those without a control group (non-cancer healthy population) were excluded. Studies that presented exclusively without full-text publication, with inappropriate raw data, or as conference abstracts were excluded. When the same study population was used in different publications, we selected the study with the largest number of participants.

### Data extraction

General information was collected from each study (first author’s last name, publication year, geographical, and clinical setting), study characteristics (study period, design, and inclusions/exclusions), and participants' characteristics (sample size, mean age, cancer type, fertility status, number of participants, control population, and adjustment factors). Serum AMH, inhibin B, basal FSH hormonal (follicular phase FSH) levels, and AFC values (mean or median and SD, SEM, 95% CI, and range) were extracted. Four reviewers (Q.Q.Z., M.W., Y.C.G,. and Y.B.H.) performed the primary evaluation by reviewing titles, abstracts, and keywords for relevance to cancer and ovarian reserve. The full text of selected articles was retrieved to provide the list of potentially eligible studies. Two authors (Q.Q.Z. and M.W.) performed the final assessment of the study eligibility. Q.Q.Z. extracted the data independently using a standard data extraction Excel form. M.W. checked the extractions for accuracy.

### Heterogeneity

Heterogeneity between studies was assessed using Cochran’s *Q* and *I*^2^ statistics (Higgins and Thompson, 2002), and *I*^2^ with values of 0%, 25%, 50%, and 75% provided evidence for no, low, moderate, and high between-heterogeneity, respectively. Sensitivity analyses were performed by excluding one study at a time to clarify whether the results were driven by one large study or a study with an extreme result. Additional sensitivity analyses were performed for subgroup analyses whenever possible to evaluate potential sources of heterogeneity based on study characteristics, such as study design (i.e. cohort, case-control, and cross-sectional), geographic location, study quality per the ROBINS-I tool (‘low’ or ‘moderate’ versus ‘serious’ or ‘critical’), cancer type or biochemical measurements of the ovarian reserve markers. The subgroup differences were examined using meta-regression. For all analyses, the impact of the study sample size was accounted for in the statistical modeling and is represented by the width of the 95% CI.

### Publication bias

The publication bias across studies was evaluated by using funnel plots for asymmetry, Egger’s, and Begg’s tests.

### Risk of bias assessment

Based on the extracted data, we used the risk of bias in non-randomized studies of interventions (ROBINS-I) tool to assess the quality of the eligible studies (Sterne *et al.*, 2016). The tool contains seven domains: bias due to confounding, bias in study participant selection, bias in exposure measurement, bias due to misclassification of exposure during follow-up, bias due to missing data, bias in outcome measurement, and bias in the selection of reported results. A study was deemed to have a low risk of bias when it was rated as probably at risk for one domain, and a study was considered at serious or critical risk of bias when rated as high risk for more than one domain or critical risk in at least one domain, respectively. ‘No information’ was applied to a study when information was missing in at least two domains. Two authors (Q.Q.Z. and Y.C.G.) performed the evaluation independently. Any disagreements were resolved via consensus or mediation by a third author (M.W.).

### Outcomes and statistical analysis

Quantitative results in all settings were extracted or converted into means and SD. If mean or SD values were unavailable and the median, SEM, range, and 95% CI were reported; we estimated the mean and SD using the formulas suggested for meta-analysis to pool data (Wan *et al.*, 2014; Luo *et al.*, 2018). When there were multiple cancer groups and a single control group, the age between the two independent case–control groups was compared using Student’s *t* test. For continuous variables with consistent units, including inhibin B, basal FSH, and AFC, we calculated the weighted mean difference (WMD) with the associated 95% CIs between the study and control groups for the effect of cancer on ovarian reserve in the meta-analysis. For AMH variables with inconsistent units, we calculated the standard mean difference (SMD). Fixed or random effects meta-analysis was used according to the heterogeneity of pooled data. The statistical analyses were performed using Stata software (Stata Corp, College Station, TX, USA).

## Results

### Study selection

**Figure 1.**
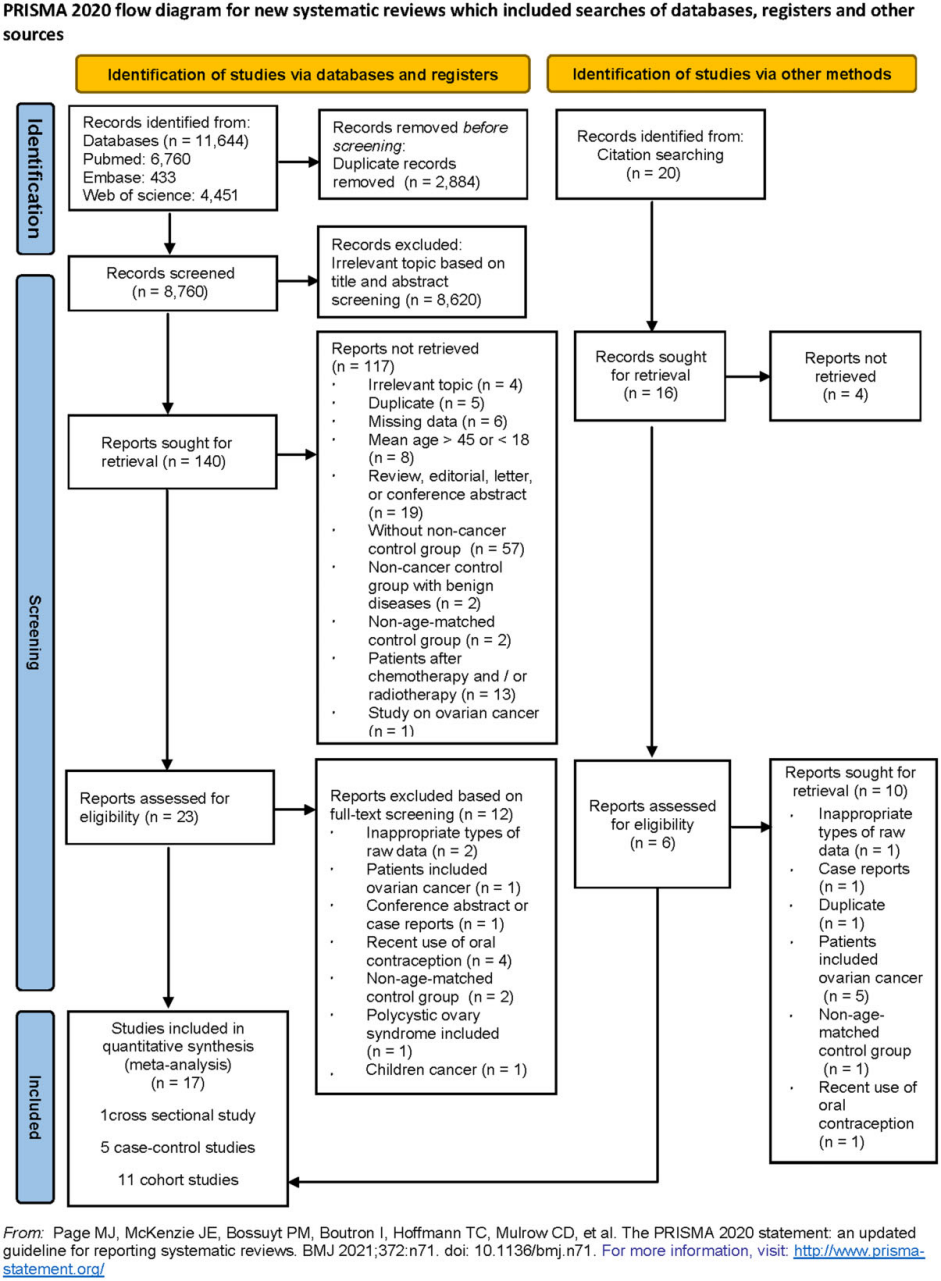
Flow chart of study selection for a systematic review and meta-analysis of cancer and ovarian reserve.

The initial search identified 11 644 records: including 6760 from PubMed, 433 from Embase, 4451 from Web of Science, and 20 from additional records from other sources. After excluding studies that did not meet the inclusion criteria, a total of 17 publications (11 cohort, 5 case–control, and 1 cross-sectional studies) were considered eligible for quantitative synthesis (Oktay *et al.*, 2006; Lutchman Singh *et al.*, 2007; Lie Fong *et al.*, 2008; Quintero *et al.*, 2010; Yu *et al.*, 2010; Das *et al.*, 2011; Johnson *et al.*, 2013; Bala *et al.*, 2016; Naasan *et al.*, 2016; Paradisi *et al.*, 2016a,b; Pereira *et al.*, 2016; Quinn *et al.*, 2017; Decanter *et al.*, 2018; Goldrat *et al.*, 2019; Gunnala *et al.*, 2019; Porcu *et al.*, 2020) (Fig. 1). The characteristics and results of the included studies are presented in Supplementary Table S2.

### Study characteristics

The 17 studies consisted of 8150 women of reproductive age with 1197 and 6953 in the cancer and control groups, respectively. To comprehensively understand the effects of cancer on ovarian reserve, we pooled all of the literature on corresponding ovarian reserve biomarkers for an overall cancer analysis regardless of cancer type.

Of these, there were enough data for subtype analyses of breast cancer and hematological malignancies, and so the corresponding data were combined for subsequent analyses on cancer types. Eight studies related to breast cancer (Oktay *et al.*, 2006; Lutchman Singh *et al.*, 2007; Yu *et al.*, 2010; Bala *et al.*, 2016; Pereira *et al.*, 2016; Quinn *et al.*, 2017; Goldrat *et al.*, 2019; Porcu *et al.*, 2020), with four studies reporting serum AMH levels (Yu *et al.*, 2010; Bala *et al.*, 2016; Pereira *et al.*, 2016; Goldrat *et al.*, 2019), two studies separately reporting basal FSH hormone levels and AFC values (Oktay *et al.*, 2006; Quinn *et al.*, 2017), and two studies reporting multiple ovarian reserve parameters (e.g. AMH, inhibin B, and AFC) (Lutchman Singh *et al.*, 2007; Porcu *et al.*, 2020). Hematological malignancies were analyzed in another two studies (Lie Fong *et al.*, 2008; Paradisi *et al.*, 2016a,b), both of which reported serum AMH levels. Three additional studies evaluated overall cancers without describing the cancer type in detail (Johnson *et al.*, 2013; Paradisi *et al.*, 2016a,b; Decanter *et al.*, 2018), with AMH and AFC levels analyzed. Another three studies focused on multiple cancer types (e.g. breast cancer, endometrial cancer, brain cancer, and bone cancer) (Das *et al.*, 2011; Naasan *et al.*, 2016; Gunnala *et al.*, 2019), for which serum AMH, basal FSH, and AFC were reported, respectively. One particular study reported on both overall cancer and breast cancer (Quintero *et al.*, 2010), and basal FSH levels for each were analyzed.

### Quality of evidence and risk of bias assessment

According to the ROBINS-I tool, three studies had a low risk of bias (Das *et al.*, 2011; Paradisi *et al.*, 2016a,b; Decanter *et al.*, 2018), five studies had a moderate risk of bias (Lutchman Singh *et al.*, 2007; Bala *et al.*, 2016; Naasan *et al.*, 2016; Paradisi *et al.*, 2016a,b; Pereira *et al.*, 2016), and nine studies had a serious risk of bias (Oktay *et al.*, 2006; Lie Fong *et al.*, 2008; Quintero *et al.*, 2010; Yu *et al.*, 2010; Johnson *et al.*, 2013; Quinn *et al.*, 2017; Goldrat *et al.*, 2019; Gunnala *et al.*, 2019; Porcu *et al.*, 2020) (Table 1).

**Table 1.** Risk of bias for the 17 included studies (2006–2020) based on the ROBINS-I tool (low, moderate, serious, and critical).

Author, year, location	Type of bias							Overall rating
	Bias due to confounding	Bias due to selection of participants	Bias due to exposure assessment	Bias due to misclassification during follow-up	Bias due to missing data	Bias due to measurement of the outcome	Bias due to selective reporting of the results	
Oktay *et al.*, 2006, USA	Serious	Low	Low	Serious	Low	Moderate	Serious	Serious
Lutchman Singh *et al.*, 2007, UK	Low	Low	Low	Low	Moderate	Low	Moderate	Moderate
Lie Fong *et al.*, 2008, The Netherlands	Serious	Low	Serious	Serious	Serious	Moderate	Moderate	Serious
Quintero *et al.*, 2010, USA	Serious	Low	Low	Serious	Low	Low	Moderate	Serious
Yu *et al.*, 2010, USA	Serious	Low	Serious	Low	Low	Low	Moderate	Serious
Das *et al.*, 2011, Canada	Low	Low	Low	Low	Low	Low	Low	Low
Johnson *et al.*, 2013, USA	Serious	Low	Serious	Moderate	Low	Low	Moderate	Serious
Bala *et al.*, 2016, India	Moderate	Low	Low	Low	No information	Moderate	Moderate	Moderate
Naasan *et al.*, 2016, Ireland	Moderate	Low	Low	Low	Low	Low	Moderate	Moderate
Paradisi *et al.*, 2016a, Italy	Moderate	Low	Moderate	Low	No information	Low	Moderate	Moderate
Paradisi *et al.*, 2016b, Italy	Low	Low	Low	Low	Low	Low	Low	Low
Pereira *et al.*, 2016, USA	Low	Low	Low	Low	No information	Low	Moderate	Moderate
Quinn *et al.*, 2017, USA	Serious	Serious	Low	Moderate	Low	Moderate	Moderate	Serious
Decanter *et al.*, 2018, France	Low	Low	Low	Low	Low	Low	Low	Low
Goldrat *et al.*, 2019, Belgium	Serious	Low	Low	Moderate	Low	Low	Moderate	Serious
Gunnala *et al.*, 2019, USA	Serious	Low	Serious	Moderate	Low	Low	Moderate	Serious
Porcu *et al.*, 2020, Italy	Serious	Low	Low	Serious	Low	Low	Moderate	Serious

ROBINS-I tool, risk of bias in non-randomized studies of interventions tool.

### Pooled AMH results and heterogeneity analyses

**Figure 2.**
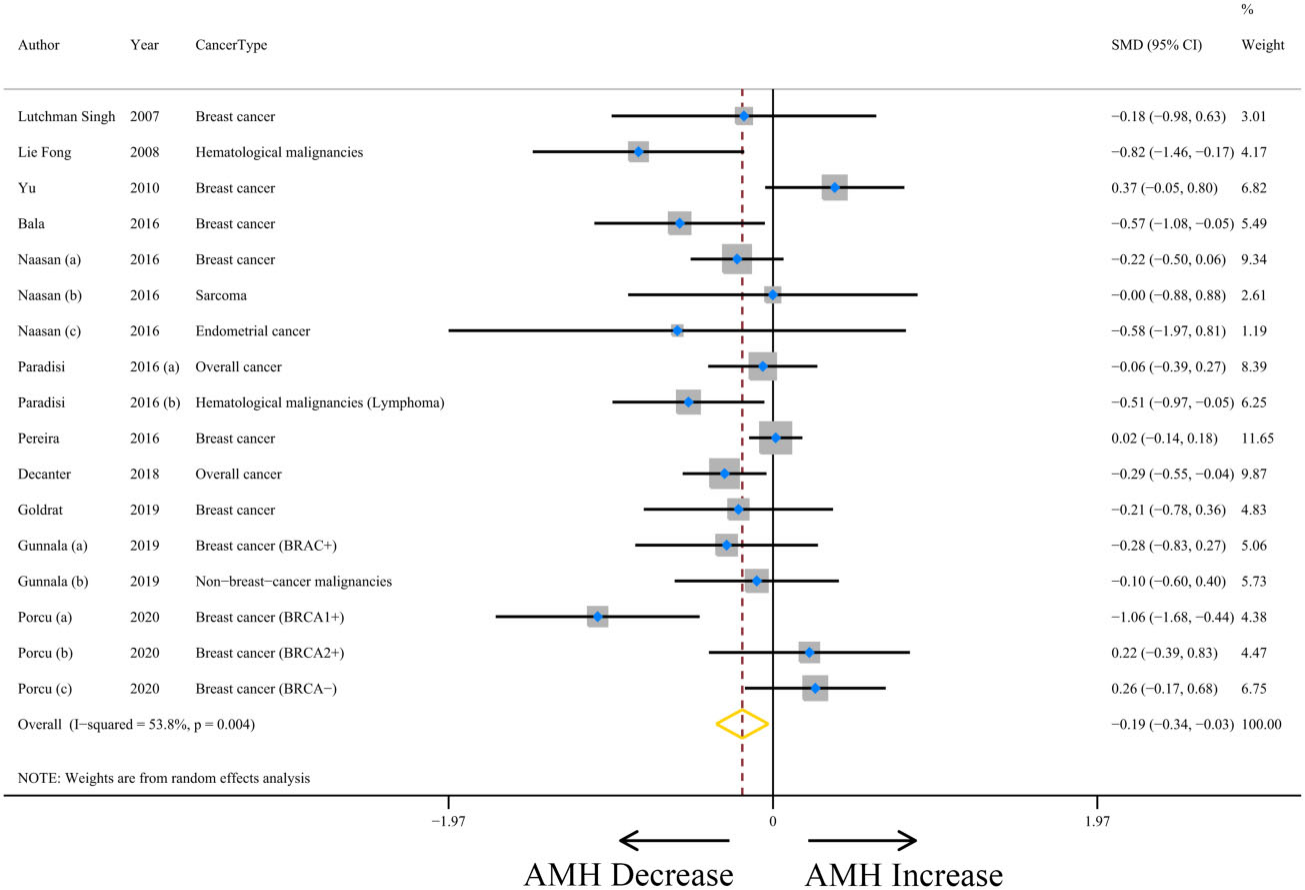
**The association between cancer and serum anti-Müllerian hormone (AMH) levels.** WMD, weighted mean difference.

The meta-analysis included 12 studies that evaluated the association between cancer and serum AMH levels (Lutchman Singh *et al.*, 2007; Lie Fong *et al.*, 2008; Yu *et al.*, 2010; Bala *et al.*, 2016; Naasan *et al.*, 2016; Paradisi *et al.*, 2016a,b; Pereira *et al.*, 2016; Decanter *et al.*, 2018; Goldrat *et al.*, 2019; Gunnala *et al.*, 2019; Porcu *et al.*, 2020) (Supplementary Table S3). For overall cancer, serum AMH levels, in 17 analyses, were lower overall compared to that of age-matched control participants (SMD = −0.19, 95% CI = −0.34 to −0.03, *P* = 0.021). However, there was moderate heterogeneity among the included studies (*I*^2^ = 53.8%, *P* = 0.004) (Fig. 2 and Table 2). We carried out subgroup analyses to detect the source of heterogeneity. However, this heterogeneity was not explained by cohort (SMD = −0.09, 95% CI = −0.31 to 0.13, *I*^2^ = 63.8%, *P* = 0.005), case–control (SMD = −0.32, 95% CI = −0.51 to −0.12, *I*^2^ = 17.1%, *P* = 0.300), or cross-sectional study design (SMD = −0.18, 95% CI = −0.98 to 0.63; *P*_heterogeneity_ = 0.223). The magnitude of heterogeneity was low when stratified by geographic location and cancer type except for studies of breast cancer (SMD = −0.13, 95% CI = −0.35 to 0.10, *I*^2^ = 62.2%, *P* = 0.005). There was a moderate level of heterogeneity within the categories of the AMH assay (IOT: SMD = −0.54, 95% CI = −1.16 to 0.08; DSL: SMD = 0.37, 95% CI = −0.05 to 0.80; AMH Gen II: SMD = −0.27, 95% CI = −0.43 to −0.11; DSL and AMH Gen II: SMD = −0.21, 95% CI = −0.47 to 0.05; Not reported: SMD = −0.11, 95% CI = −0.47 to 0.25; *P*_heterogeneity_ = 0.500). The observed heterogeneity could not be eliminated by the two ROBINS-I risk of bias categories (low or moderate: SMD = −0.19, 95% CI = −0.34 to −0.04; serious or critical: SMD = −0.17, 95% CI = −0.51 to 0.17; *P*_heterogeneity_ = 0.656) (Supplementary Table S4). No statistically significant heterogeneity was found through the above subgroup analyses. We speculated that control groups with infertility history (e.g. tubal and/or idiopathic factor) may explain the potential heterogeneity to some extent (Naasan *et al.*, 2016; Goldrat *et al.*, 2019). The influence analysis generated stable summary estimates without detecting influential publications (Supplementary Fig. S1). There was no evidence of publication bias causing overestimation of the association between the cancer effect and AMH values (Supplementary Fig. S2), as shown by Egger’s (*P* = 0.172) and Begg’s test (*P* = 0.303).

**Table 2.** Results of the meta-analysis examining the association between cancer and ovarian reserve outcome measures.

	Overall cancer				Breast cancer				Hematological malignancies			
	n^a^	Effect estimate	*P*	Heterogeneity	n^a^	Effect estimate	*P*	Heterogeneity	n^a^	Effect estimate	*P*	Heterogeneity
		(95% CI)		*I* ^2^, *P*^b^		(95% CI)		*I* ^2^, *P*^b^		(95% CI)		*I* ^2^, *P*^b^
AMH^c^	**17**	−**0.19 (**−**0.34,** −**0.03)^R^**	**0.021**	**53.8%, 0.004**	10	−0.13 (−0.35, 0.10)^R^	0.266	62.2%, 0.005	**2**	−**0.62 (**−**0.99,** −**0.24)**	**0.001**	**0.0%, 0.452**
Inhibin B^d^	2	−5.35 (−19.16, 8.47)	0.448	0.0%, 0.876	NC	NC	NC	NC	NC	NC	NC	NC
Basal FSH^d^	11	0.00 (−0.85,0.85)^R^	0.997	79.3%, 0.000	7	0.21 (−0.99, 1.41)^R^	0.727	84.1%, 0.000	NC	NC	NC	NC
AFC^d^	**16**	−**0.93 (**−**1.79,** −**0.07)**	**0.033**	37.4%, 0.066	9	−0.66 (−1.72, 0.41)	0.225	0.0%, 0.842	NC	NC	NC	NC

Bold records denote statistically significant associations. All pooled effect estimates are derived from fixed-effects analyses, except for records marked with ^R^(random-effects).

AMH, anti-Müllerian hormone; FSH, follicle-stimulating hormone; AFC, antral follicle count; CI, confidence interval; NC, not calculated.

^a^Number of publications.

^b^ *P*-value derived from Cochran *Q* statistic.

^c^All analyses were based on SMD.

^d^All analyses were based on WMD.

Because there was a significant difference between the standard mean AMH of overall cancer and healthy controls, we examined which type of cancer contributed to the decreased AMH value. Using the control individuals as a reference, the SMD in breast cancer was −0.13 (95% CI = −0.35 to 0.10, *P* = 0.266), with moderate heterogeneity (*I*^2^ = 62.2%, *P* = 0.005) (Table 2; Supplementary Fig. S3). Patients with hematological malignancies had significantly lower levels of serum AMH, with an estimated SMD of −0.62 (95% CI = −0.99 to −0.24, *P* = 0.001) and without heterogeneity (*I*^2^ = 0.0%, *P* = 0.452) (Table 2; Supplementary Fig. S4). The results show that breast cancer may not damage ovarian function, but hematological malignancies may lead to lower levels of AMH. However, further studies are needed to investigate the possible impact of cancer type and stage on the level of AMH.

### Pooled inhibin B results

The analysis of the association between cancer and inhibin B levels included two studies (Lutchman Singh *et al.*, 2007; Paradisi *et al.*, 2016a,b) (Supplementary Table S3). There was no significant difference in serum inhibin B levels between patients with cancer and healthy controls with a WMD of −5.35 (95% CI = −19.16 to 8.47, *P* = 0.448). There was no evidence of heterogeneity (*I*^2^ = 0.0%, *P* = 0.876) (Table 2; Supplementary Fig. S5). No testing for publication bias was performed because of the small number of included studies.

### Pooled basal FSH results and heterogeneity analyses

**Figure 3.**
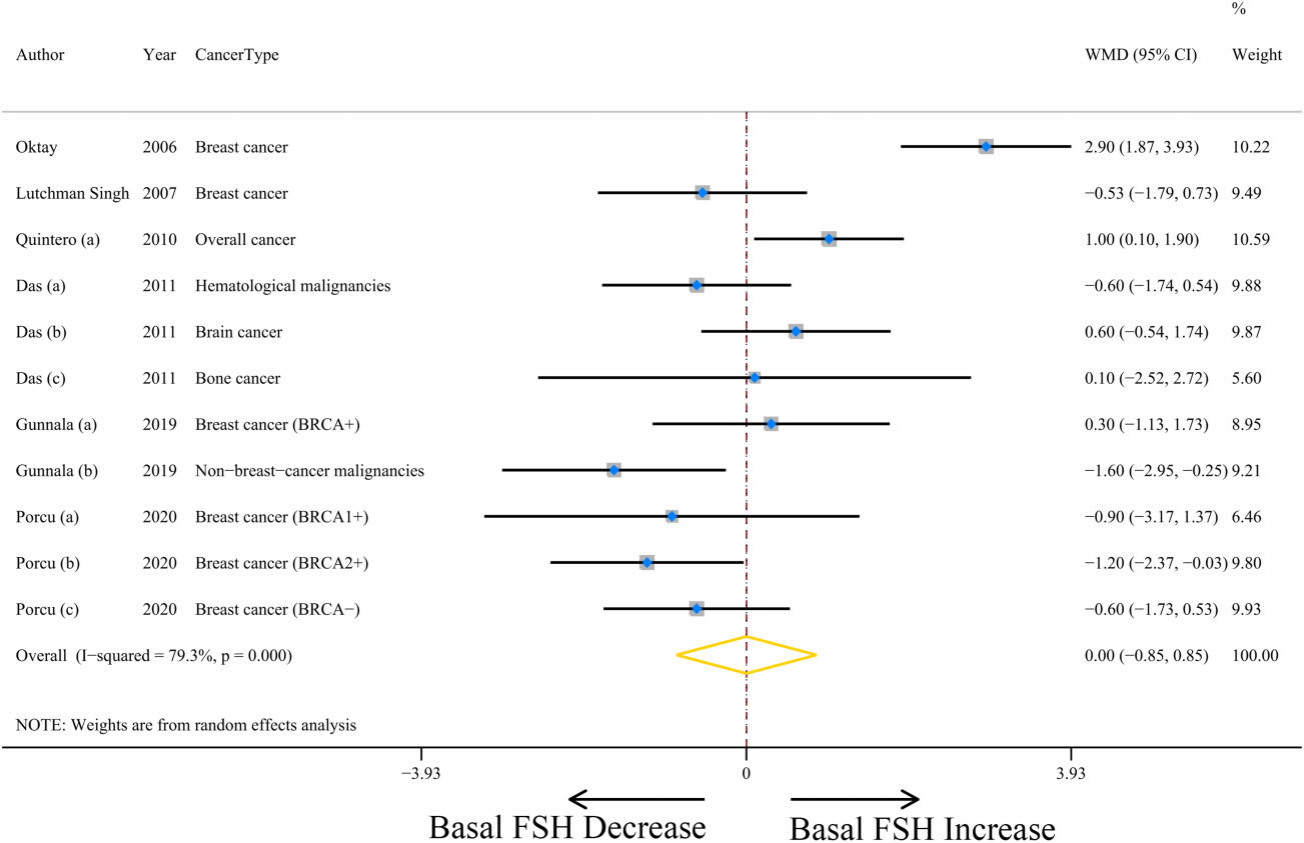
**The association between cancer and serum basal FSH.** WMD, weighted mean difference.

A total of six studies were included in the analysis of basal FSH levels (Oktay *et al.*, 2006; Lutchman Singh *et al.*, 2007; Quintero *et al.*, 2010; Das *et al.*, 2011; Gunnala *et al.*, 2019; Porcu *et al.*, 2020) (Supplementary Table S3), and the weighted mean basal FSH values were not statistically significant between the cancer patients and control groups (WMD = 0.00, 95% CI = −0.85 to 0.85, *P* = 0.997) (Fig. 3). Heterogeneity between the studies was high (*I*^2^ = 79.3%, *P* = 0.000) (Table 2). There was a high level of heterogeneity within study designs, geographic location, ROBINS-I risk of bias, and cancer type (Supplementary Table S4), while no influential studies were found based on sensitivity analyses (Supplementary Fig. S6). There was no publication bias in eligible studies (all Egger’s or Begg’s test *P* > 0.28; Supplementary Fig. S7).

Next, we performed subgroup analyses to find the association between types of cancer and basal FSH. Four studies focused on breast cancer were included in the analysis (Oktay *et al.*, 2006; Lutchman Singh *et al.*, 2007; Quintero *et al.*, 2010; Porcu *et al.*, 2020). A non-statistically significant WMD of 0.21 (95% CI = −0.99 to 1.41, *P* = 0.727) was found for breast cancer (Supplementary Fig. S8). Heterogeneity between these studies was high (*I*^2^ = 84.1%, *P* = 0.000) (Table 2).

### Pooled AFC results

**Figure 4.**
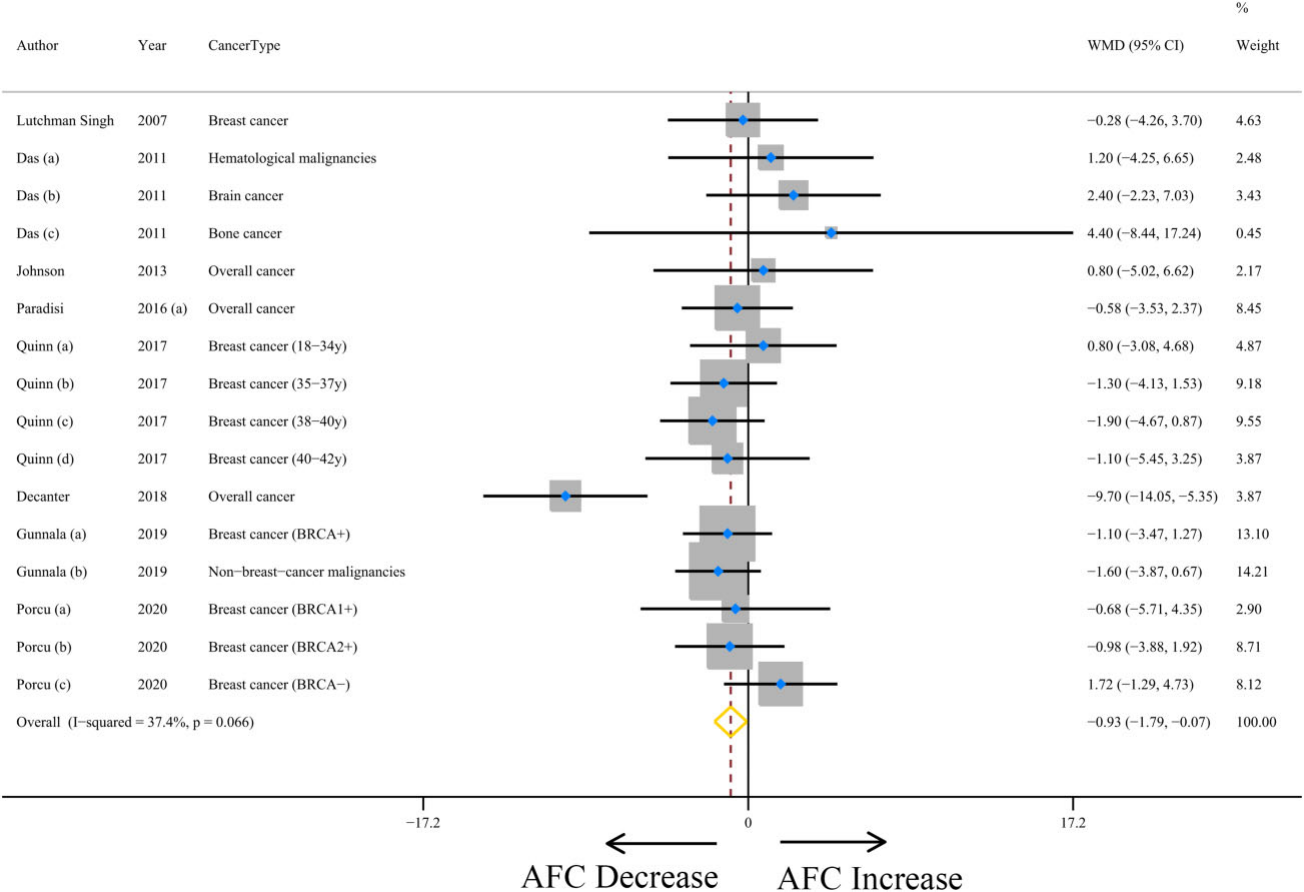
**The association between cancer and antral follicle count (AFC).** WMD, weighted mean difference.

Eight studies evaluating the impact of cancer on AFC values were included (Lutchman Singh *et al.*, 2007; Das *et al.*, 2011; Johnson *et al.*, 2013; Paradisi *et al.*, 2016a,b; Quinn *et al.*, 2017; Decanter *et al.*, 2018; Gunnala *et al.*, 2019; Porcu *et al.*, 2020) (Supplementary Table S3). When compared to controls, cancer patients had significantly lower weighted mean AFC values (WMD: −0.93, with 95% CI = −1.79 to −0.07; *P* = 0.033) (Fig. 4 and Table 2) with slight level of heterogeneity for this comparison (*I*^2^ = 37.4%, *P* = 0.066). No evidence of publication bias was found by Egger’s test (*P* = 0.524), although moderate publication bias was found by Begg’s test (*P* = 0.034) (Supplementary Fig. S9).

For the cancer type categories, there was an indication of a lower tendency in the mean AFC in patients with breast cancer compared with the controls, with a non-statistically significant difference (WMD: −0.66, with 95% CI = −1.72 to 0.41; *P* = 0.225). There was no evidence of heterogeneity (*I*^2^ = 0.0%, *P* = 0.842) (Table 2; Supplementary Fig. S10).

## Discussion

### Principal findings

Previous publications have revealed conflicting results when examining the association between cancer and ovarian reserve prior to therapy. To our knowledge, this study is the first comprehensive meta-analysis investigating ovarian reserve in patients with cancer before gonadal toxicity treatment. Collectively, the eligible studies include a large sample size of population, including 8150 women of reproductive age, with 1197 and 6953 in the cancer and control groups, respectively. The present study revealed that patients with cancer had significant decreased serum AMH levels and AFC values before cancer treatment, especially patients with hematological malignancies. Basal FSH levels and inhibin B levels showed no difference between cancer patients and control groups. These findings, which need to be confirmed in larger sample sizes, should be considered and may influence the oncofertility counseling of this specific patient population.

### Comparison with existing literature

Breast cancer is the most common malignancy in women of reproductive age (Pashayan *et al.*, 2020), and we investigated the association between breast cancer and ovarian reserve. Seven studies reported no significantly differences in AMH and AFC levels in patients with breast cancer (Lutchman Singh *et al.*, 2007; Yu *et al.*, 2010; Naasan *et al.*, 2016; Pereira *et al.*, 2016; Quinn *et al.*, 2017; Goldrat *et al.*, 2019; Gunnala *et al.*, 2019). Conversely, only one study reported significantly lower AMH levels (Bala *et al.*, 2016). Our meta-analysis also found that women with breast cancer showed no significant difference in serum AMH levels and AFC values compared with healthy controls. There were two studies that showed higher basal FSH levels (Oktay *et al.*, 2006; Quintero *et al.*, 2010), while the other two reported no difference in patients with breast cancer (Lutchman Singh *et al.*, 2007; Gunnala *et al.*, 2019). We revealed that the basal FSH levels also showed no significant difference between patients with breast cancer and healthy controls. Hence, our results revealed that breast cancer has no negative impact on ovarian reserve, while more original studies are needed to confirm this conclusion. Interestingly, an included study by Porcu et al. (2020) reported that breast cancer patients with *BRCA1* mutations, but not *BRCA2* mutations, had significantly lower AMH levels. Some studies suggested that *BRCA1* or *BRCA2* mutations were involved in infertility or POI (Oktay *et al.*, 2010; Titus *et al.*, 2013; Wang *et al.*, 2014; Lambertini *et al.*, 2018; Son *et al.*, 2019; Turan *et al.*, 2021). Therefore, it can be hypothesized that the missing *BRCA1* repair proteins dramatically prevent breast cancer patients having enough ovarian reserve to enable a potential conception. However, due to the small number of articles, a meta-analysis on the relationship between *BRCA* mutations, breast cancer and ovarian reserve was not possible.

In recent decades, the survival rate after hematological malignancies has been improved significantly, but ovarian function is negatively affected by the detrimental effect of hematological malignancies on follicular health before the administration of chemotherapy or radiotherapy (Lie Fong *et al.*, 2008; Lawrenz *et al.*, 2012; van Dorp *et al.*, 2014; Paradisi *et al.*, 2016a,b). Lie Fong et al. (2008) and Paradisi *et al.* (2016a,b) reported lower AMH levels in patients with hematological malignancies before treatment compared to healthy controls. Patients with lymphoma had much lower AMH and AFC levels than healthy controls and patients with other malignancies (Lekovich *et al.*, 2016). Another study also showed decreased serum AMH levels in pubertal girls with newly diagnosed leukemia and lymphoma before treatment (van Dorp *et al.*, 2014). Higher concentrations of proinflammatory cytokines may lead to the reduction of ovarian reserve in lymphoma patients (Paradisi *et al.*, 2016a,b). Similarly, our study revealed that AMH levels in patients with hematological malignancies were significantly lower than in controls. These findings highlighted that patients with hematological malignancies experience decreased AMH levels, although more studies should be performed to confirm this conclusion.

Few studies have explored other types of cancers and ovarian reserve, and some studies have analyzed the relationship between overall cancers and ovarian reserve (Das *et al.*, 2011; Paradisi *et al.*, 2016a,b; Dolinko *et al.*, 2018). Das et al. (2011) and Dolinko *et al.* (2018) found no significant difference in AFC levels between women with overall cancers (including breast cancer, gynecological cancer, hematologic cancer, gastrointestinal cancer, brain cancer, and bone cancer) and their control groups. Johnson et al. (2013) also found that cancer patients and control subjects did not differ in ovarian reserve markers. In contrast, a decreased serum AMH concentration was shown in women with gliomas (Nordan *et al.*, 2020). Therefore, further original studies are needed to determine whether there is a difference in ovarian reserve between women with other types of cancers and healthy controls.

The reason for the decrease of ovarian reserve in patients with cancer is not clear. Previous studies have indicated that cancer was often characterized by anorexia, an increased catabolic state and malnutrition which results in weight loss (Chapman *et al.*, 2009; Kuokkanen *et al.*, 2016). Meczekalski *et al.* (2006) showed lower AMH levels in patients with weight loss-related amenorrhea. Cancer-associated stress hormone secretion is also responsible for a depleted ovarian reserve (Valsamakis *et al.*, 2019). Another potential cause of the decreased ovarian reserve may be impaired DNA repair mechanisms. Genetic variation in DNA repair genes is associated with cancer and ovarian aging (Turan and Oktay, 2020). *BRCA* mutations increase the risk of breast malignancy and ovarian cancer (Roy *et al.*, 2011). Recent laboratory data also indicate that patients with *BRCA* mutations, especially *BRCA1* mutations, generally have reduced AMH concentrations (Daum *et al.*, 2018; Porcu *et al.*, 2020; Turan and Oktay, 2020). Other factors, such as immune inflammation caused by tumors, cannot be excluded. Paradisi *et al.* (2016a,b) noted that patients with lymphoma with lower AMH levels had higher concentrations of soluble IL-2 receptor (SIL-2R), IL-6, IL-8, and TNF-α compared to healthy volunteers, which indicated that cytokines may be the causal factor in the reduction of ovarian reserve. According to the conclusions reported in the literature, the tumor-associated changes in physical status, stress, genetic variation, and/or immune inflammation may explain for the decreased ovarian reserve prior to cancer treatments.

### Strengths and limitations

Our systematic review and meta-analysis is the first study targeted female ovarian reserve prior to gonadotoxic treatments, comparing patients with cancer to healthy controls, and showing significant decreases in serum AMH levels and AFC values. However, in oncological conditions, the physiology of the ovary may change. The equilibrium between the remnant follicular pool and the number of growing follicles may be tempered so that one has lower AMH or AFC, but not lower ovarian reserve. High levels of heterogeneity were found in some indexes, i.e. serum AMH and basal FSH levels. The heterogeneity remained high in AMH and basal FSH levels when subgroup analyses were delimited to studies design, geographic location, assay method, risk of bias, or cancer type. Due to the small number of studies contributing to most subgroup analyses, the power to find statistically significant heterogeneity among categories was extremely limited, which will adversely affect the current conclusions. In addition, surgery, chemotherapy, or radiotherapy treatments are required when tumor are diagnosed, so high quality randomized controlled trials or prospective cohorts about ovarian function before cancer treatment are lacking. Many of the studies included were of moderate to high risk of bias, which may lead to the high heterogeneity and imprecise results. Moreover, the number of studies for specific cancer subtypes may be too small to draw conclusions, and more studies are needed to investigate the possible impact of cancer type and stage on ovarian function.

## Conclusion

In conclusion, our study confirmed the findings that cancer negatively affects the serum AMH levels as well as the AFC values of reproductive-aged women. Based on our meta-analysis, clinicians should raise awareness about the possible need for personalized approaches for young women with cancer who are interested in pursuing fertility preservation strategies before anticancer treatments. Fertility counseling and preservation strategies should be tailored to not only patient age and the type of treatment they will receive, but also to their specific type of cancer and their associated baseline ovarian reserve.

## Supplementary Material

hoad024_Supplementary_Data

## Data Availability

All data are available in the main text or the supplementary materials.
