# Supplementary material for: Ovarian reserve in reproductive-aged patients with cancer before gonadotoxic treatment: a systematic review and meta-analysis
Source: Hum Reprod Open. 2023 May 18;2023(3):hoad024. doi: 10.1093/hropen/hoad024 (PMC10266964; doi:10.1093/hropen/hoad024)
Supplement: hoad024_Supplementary_Data [file hoad024_supplementary_data.docx]

**Supplementary Information**

**Ovarian reserve** **in** **reproductive-aged patients with cancer** **before** **gonadotoxic treatment: a systematic review and meta-analysis**

Meng Wu^1#^, Qingqing Zhu^1#^, Yibao Huang^1^, Weicheng Tang^1^, Jun Dai^1^, Yican Guo^1^, Jiaqiang Xiong^2^, Jinjin Zhang^1^, Su Zhou^1^, Fangfang Fu^1^, Mingfu Wu^1^*, Shixuan Wang^1^*.

**Affiliations:**

1. Department of Obstetrics and Gynecology, Tongji Hospital, Tongji Medical College, Huazhong University of Science and Technology, Wuhan, Hubei, China.

2. Department of Obstetrics and Gynecology, Zhongnan Hospital of Wuhan University, Wuhan, Hubei, China.

***Corresponding author:**

Shixuan Wang, PhD, Professor, Department of Obstetrics and Gynecology, Tongji Hospital, Tongji Medical College, Huazhong University of Science and Technology, 1095 Jiefang Anv. Wuhan, Hubei 430030, China; Email: [s](mailto:shixuanwang@tjh.tjmu.edu.cn)[hixuanwang@tjh.](mailto:shixuanwang@tjh.tjmu.edu.cn)[tjmu.e](mailto:shixuanwang@tjh.tjmu.edu.cn)[du.](mailto:shixuanwang@tjh.tjmu.edu.cn)[cn](mailto:shixuanwang@tjh.tjmu.edu.cn)；ORCID: [0000-0002-8610-952X](https://orcid.org/0000-0002-8610-952X).

Mingfu Wu, PhD, Professor, Department of Obstetrics and Gynecology, Tongji Hospital, Tongji Medical College, Huazhong University of Science and Technology, 1095 Jiefang Anv. Wuhan, Hubei 430030, China; Email: [wu_mingfu@t](http://wu_mingfu@tjh.tjmu.edu.cn)[jh.tjmu.edu.cn.](http://wu_mingfu@tjh.tjmu.edu.cn) ORCID: [0009-0002-4245-4791](https://orcid.org/0000-0002-8610-952X).

**^#^These authors contributed equally to this work.**

**Supplementary Figure S1:** Influence analysis of the association between cancer and serum anti-Müllerian hormone.


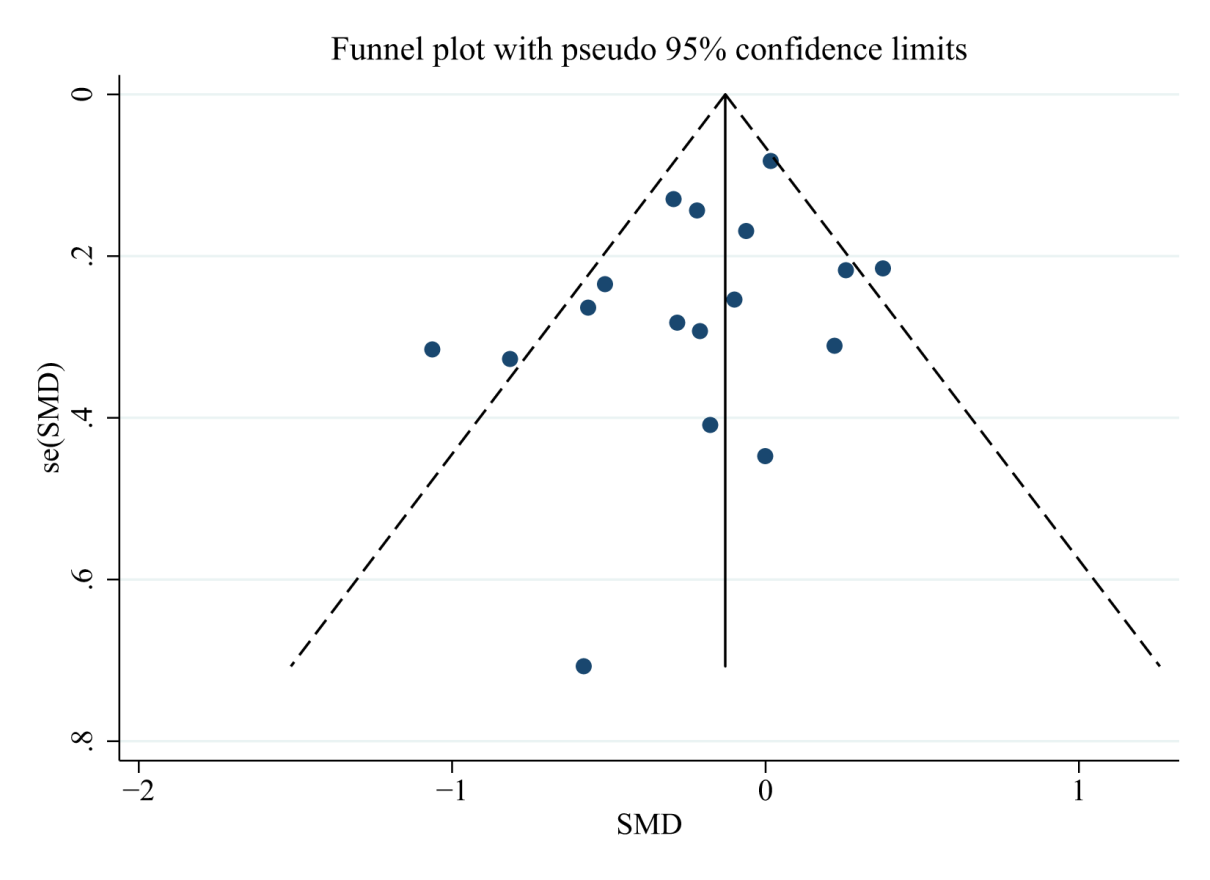


**Supplementary Figure S2:** Funnel plot for evaluating the association between cancer and serum anti-Müllerian hormone.


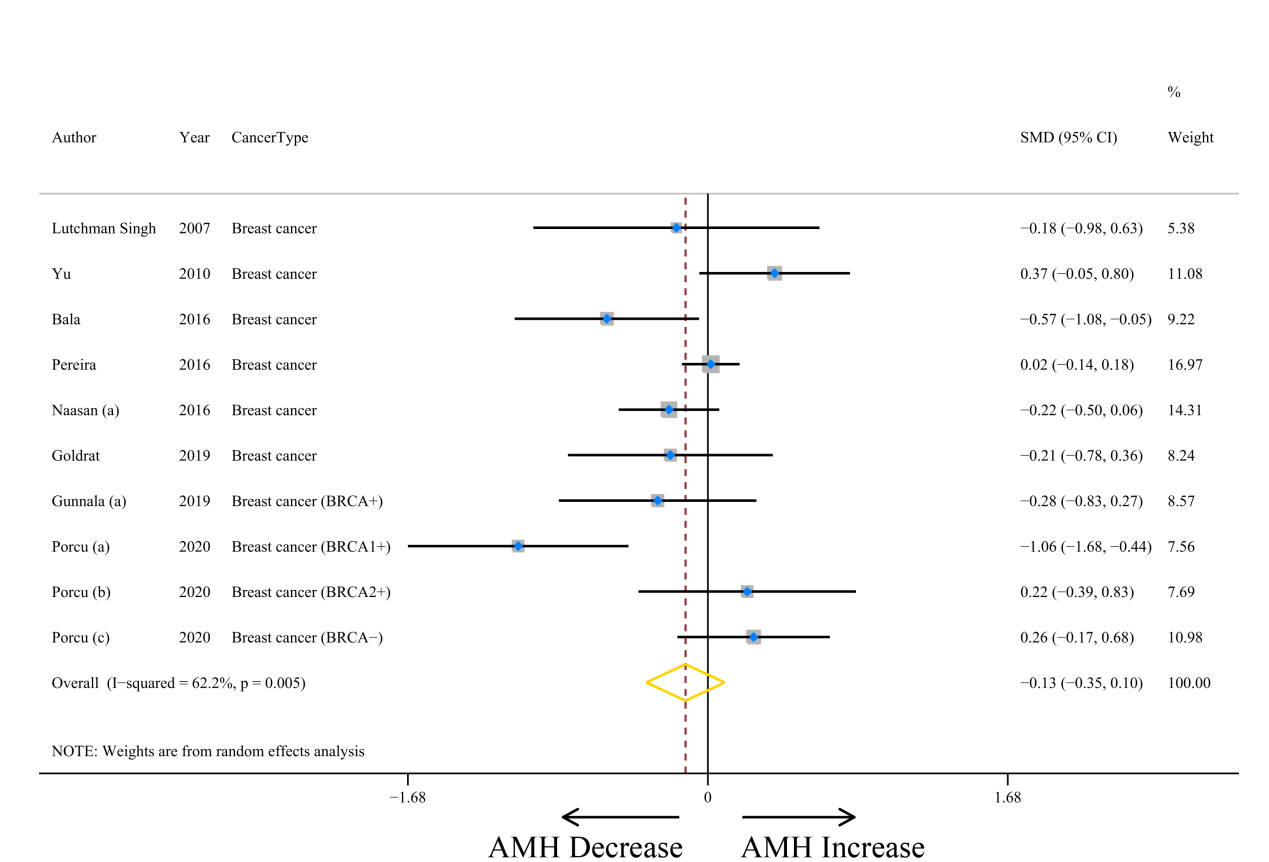
**Supplementary Figure S3**: The association between breast cancer and serum anti-Müllerian hormone.


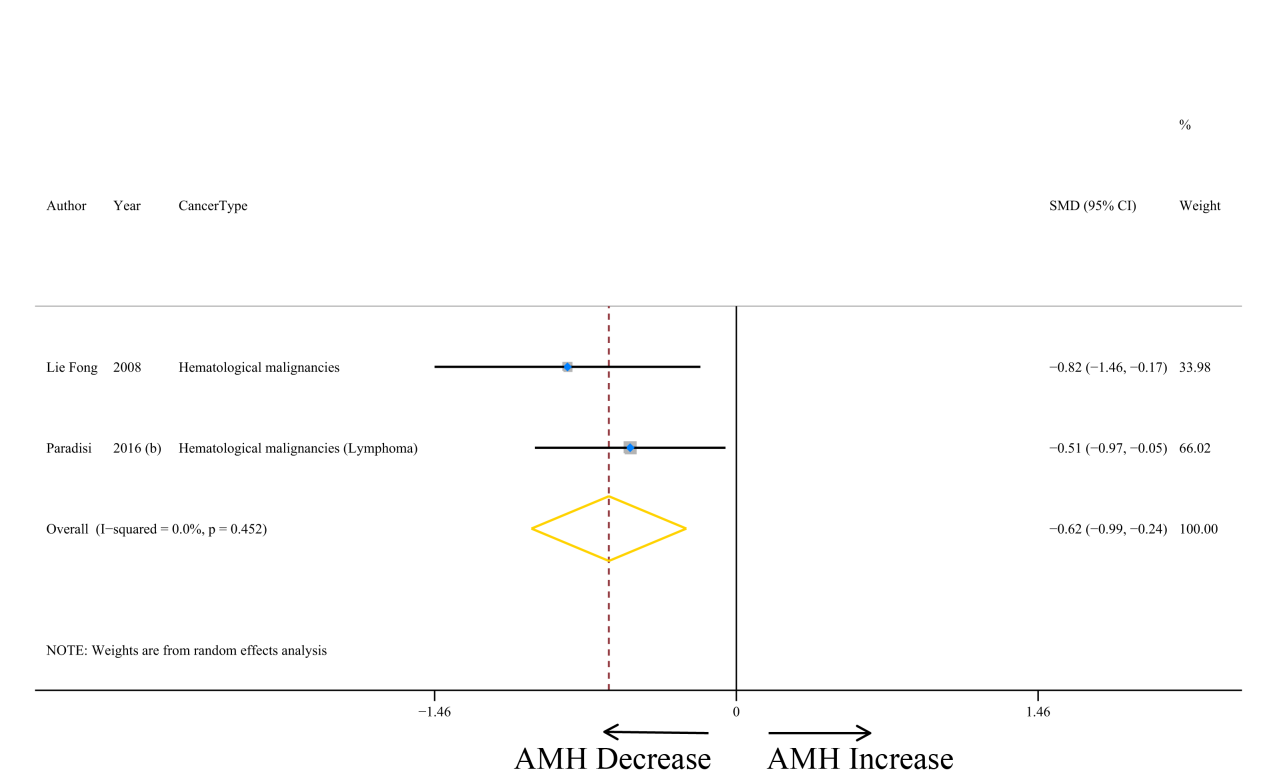


**Supplementary Figure S4:** The association between cancer and serum anti-Müllerian hormone in patients with hematological malignancies.


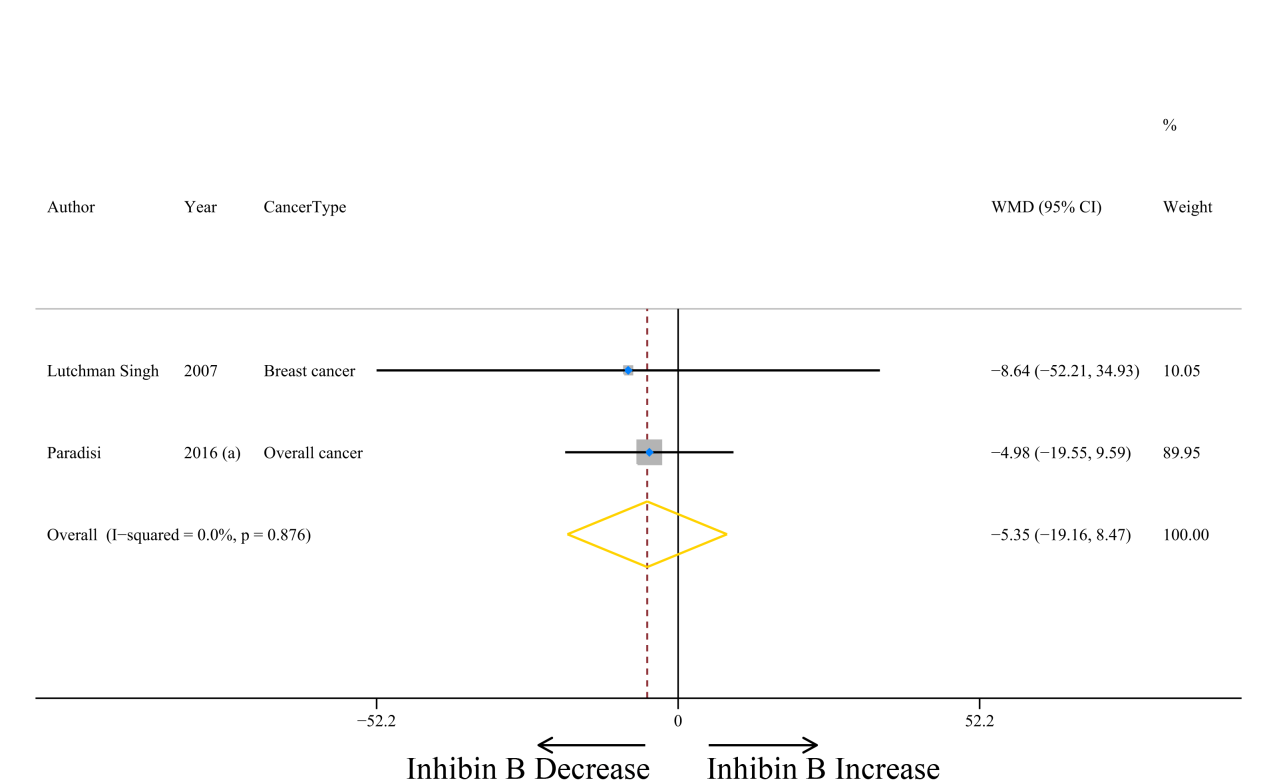


**Supplementary Figure S5:** The association between cancer and inhibin B levels.

**Supplementary Figure S6:** Influence analysis of the association between cancer and serum basal follicle-stimulating hormone.


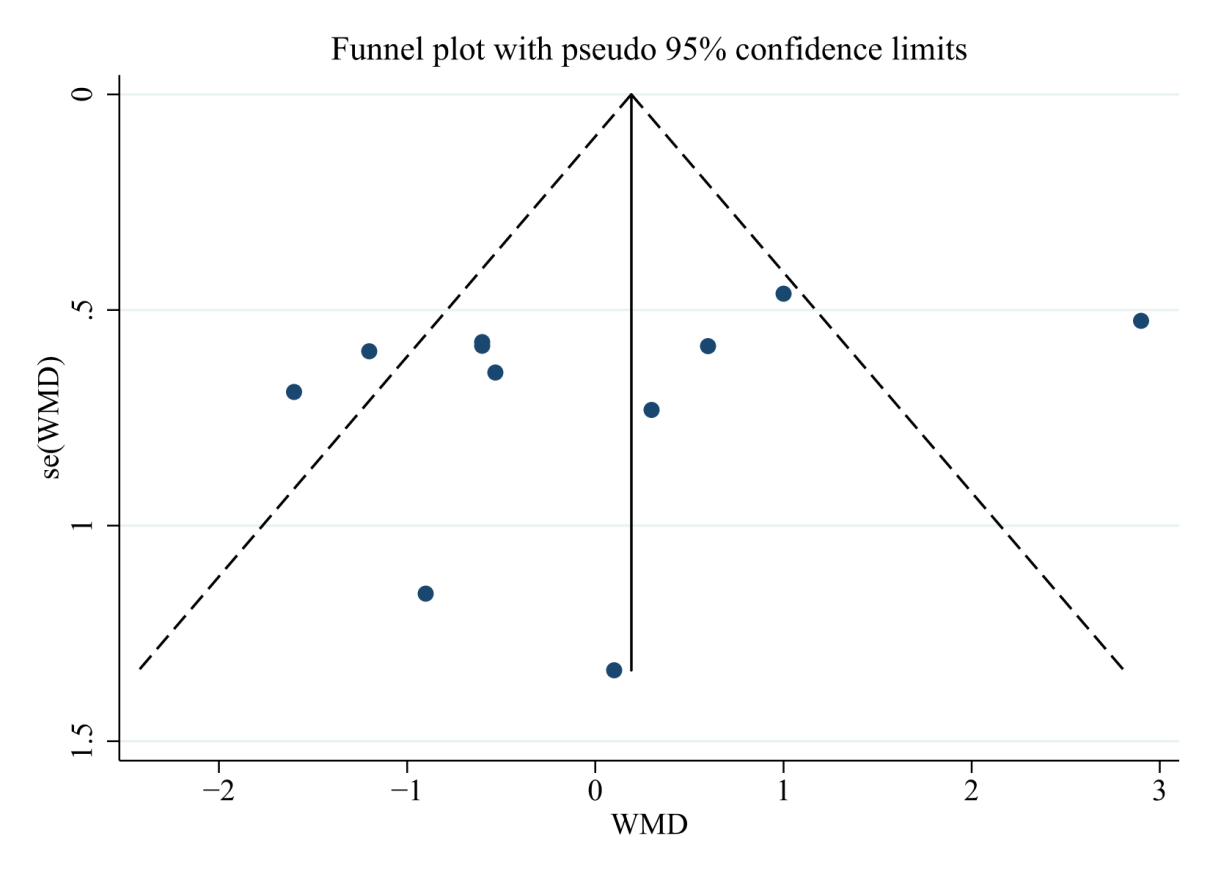


**Supplementary Figure S7:** Funnel plot evaluating the association between cancer and serum basal follicle-stimulating hormone.


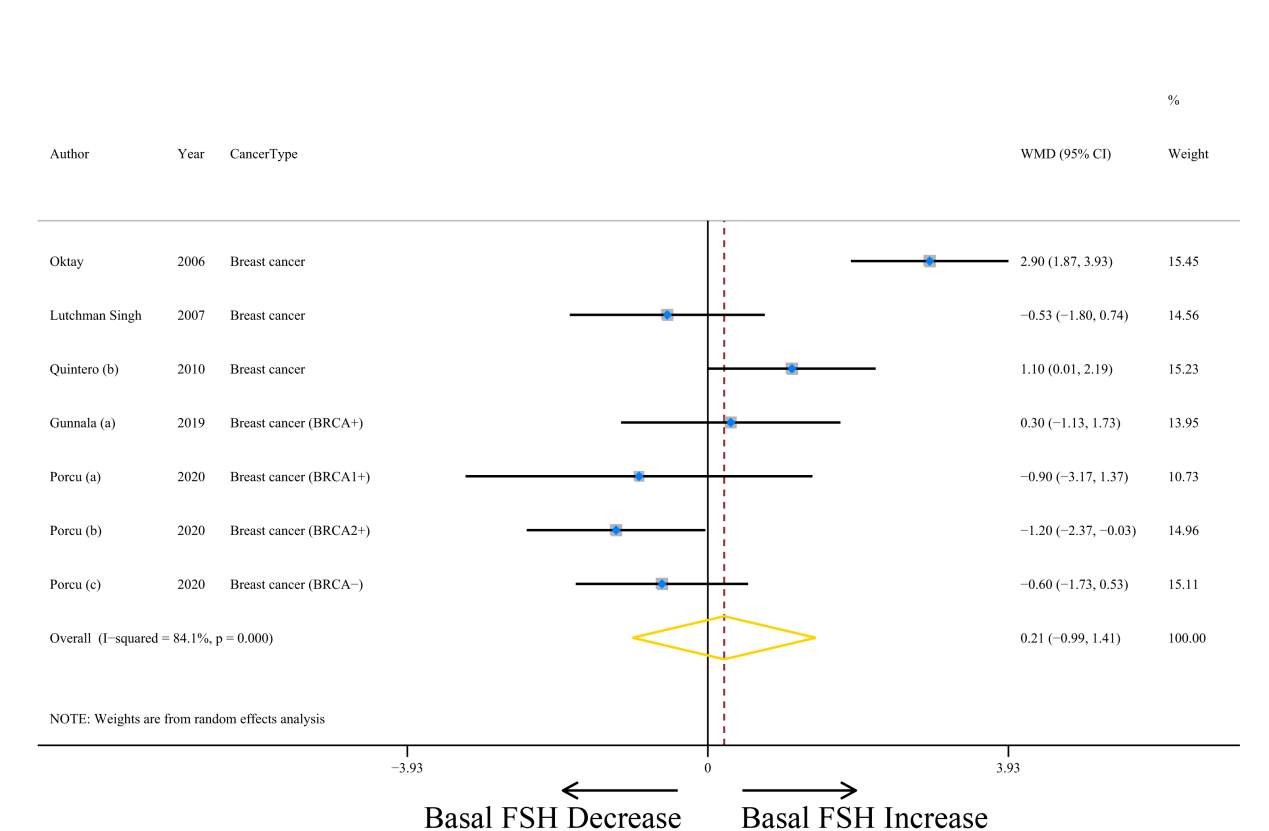


**Supplementary Figure S8:** The association between breast cancer and serum basal follicle-stimulating hormone.


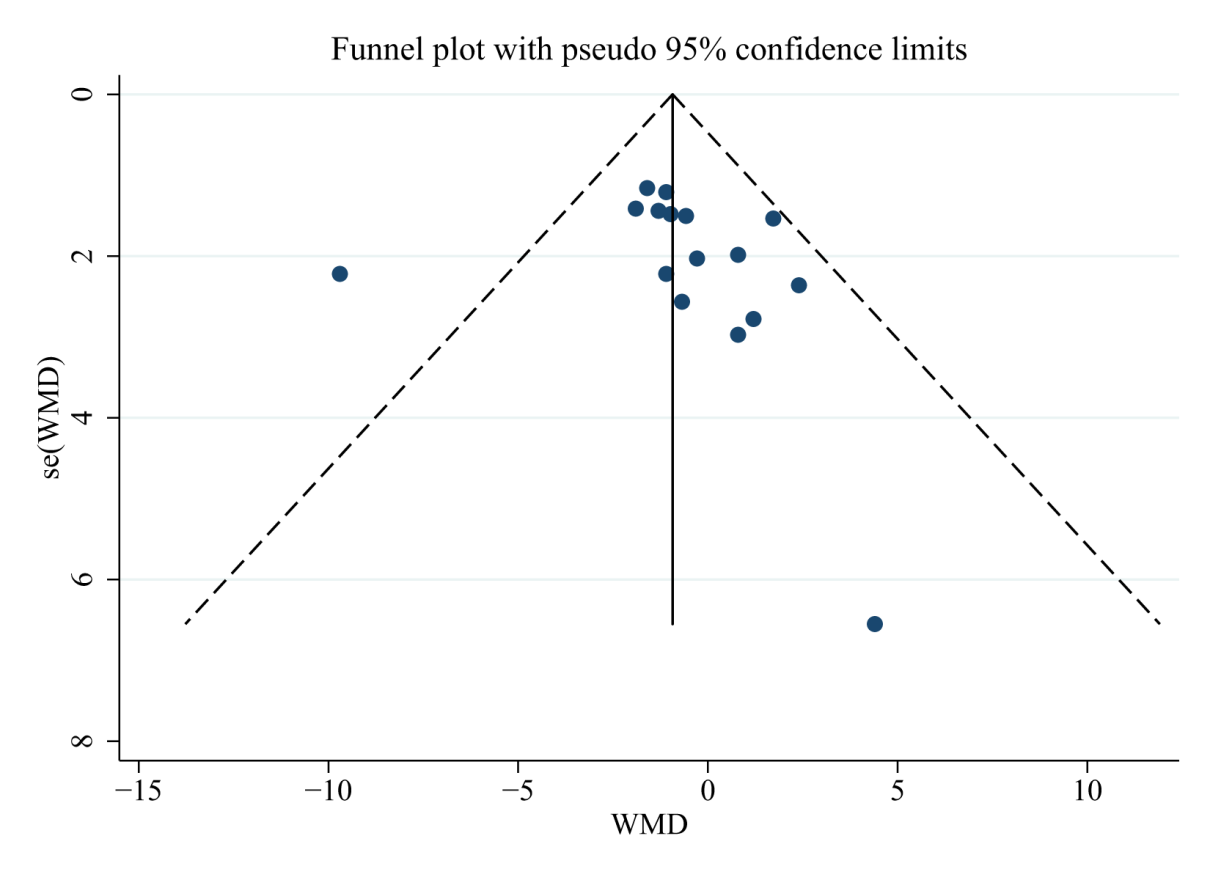


**Supplementary Figure S9:** Funnel plot evaluating the association between cancer and antral follicle count.


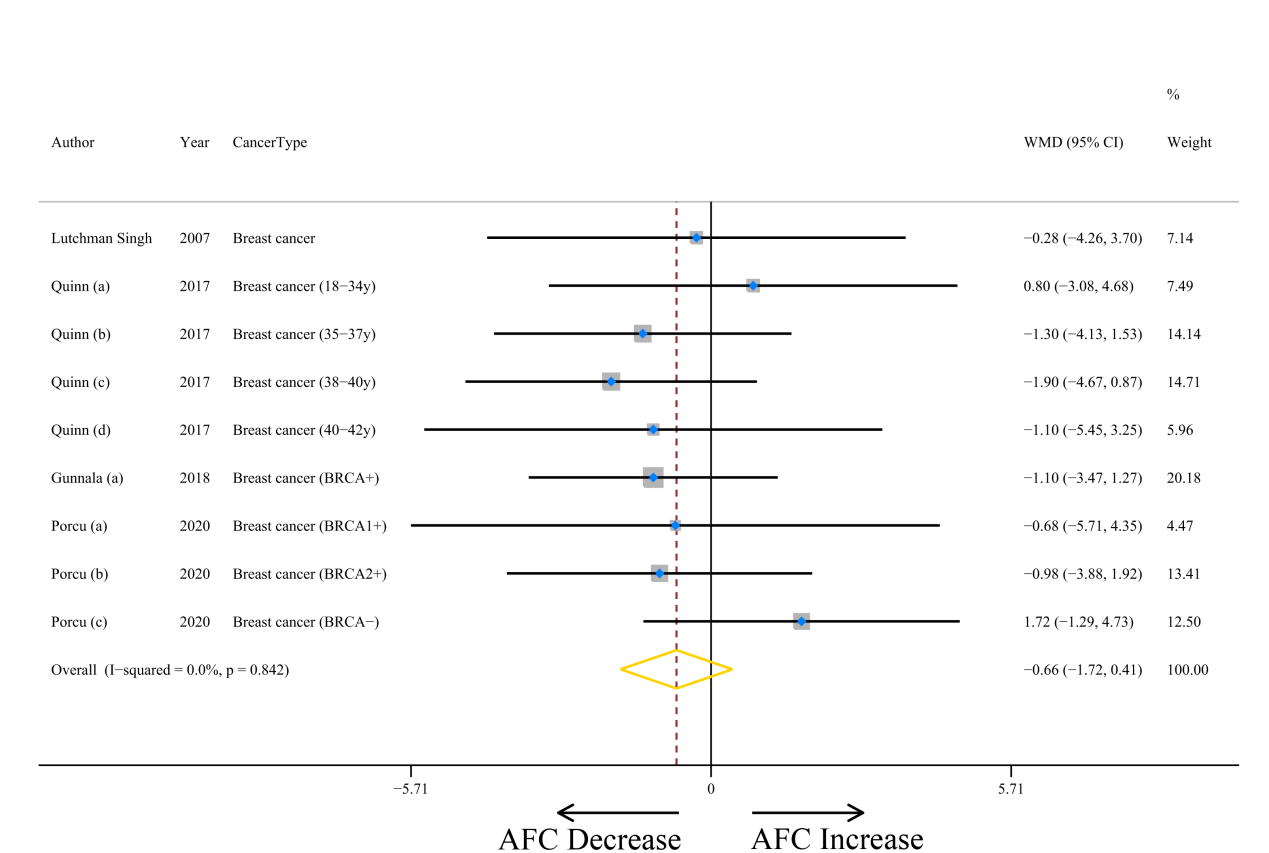


**Supplementary Figure S10:** The association between breast cancer and antral follicle count.

| **Supplementary Table S1.** Full search strategy |
| --- |
| (cancer* OR carcinoma* OR neoplasm* OR lymphoma* OR melanoma OR tumour* OR adenoma* OR sarcoma* OR tumor* OR oncolog* OR leukemi* OR leukaemi* or malignan*) AND (“ovarian function” OR “ovarian reserve” OR “ovarian reserve test” OR AMH OR “anti-Müllerian hormone” OR AFC OR “antral follicle count” OR FSH OR “follicle stimulating hormone” OR “inhibin B” OR inh B OR “ovarian volume”). |

| **Supplementary Table S2.** Characteristics of the included studies for analyses of the association between cancer and ovarian reserve function. | | | | | | | | | |
| --- | --- | --- | --- | --- | --- | --- | --- | --- | --- |
| Author  (year) | Geographic location | Study period | Study  design | Sample size | Cancer group  Included | Control group included | Exclusion criteria | ORT and ovarian stimulation response outcome measures | Adjusting factors |
| Oktay, et al.  (2006) | USA | 2003-2005 | Retrospective cohort | 103 | • Stages I-IIIA BC with delaying chemotherapy;  • IVF setting | • Age-matched tubal factor infertility  • IVF-ICSI | Stage IV BC | Basal FSH | hCG administration |
| Lutchman Singh et al.  (2007) | UK | ND | Cross sectional | 46 | • Patients with cancer before chemotherapy  • No recent hormonal therapy | • Age-matched controls without medical illness and had proven fertility  • No recent OCP | ND | AMH, Inhibin B, basal FSH, AFC | ND |
| Lie Fong et al.  (2008) | The  Netherlands | 1995-2004 | Case-control | 67 | • Patients with hematologic cancer before cancer treatments  • No OCP | • Healthy women without cancer and all proven fertile  • Age: 20 to 35  • Without endocrine disease • No hormonal therapy or OCP | ND | AMH | Age |
| Quintero, et al.  (2010) | USA | 1999-2007 | Retrospective  cohort | 100 | • Patients with cancer undergoing oocyte retrieval  • IVF setting | • Age-matched male factor infertility  • IVF setting | • Day 3 FSH ≥12 IU/mL  • Age > 40  • Received chemotherapy or pelvic radiotherapy | Basal FSH | ND |
| Yu, et al.  (2010) | USA | 2002-2006 | Prospective  cohort | 160 | • BC patients before the initiation of chemotherapy  • Age < 40 | • Age-matched healthy controls with known fertility  • Age: 30 to 40 | ND | AMH | ND |
| Das, et al.  (2011) | Canada | 2003-2010 | Retrospective cohort | 89 | • Patients with cancer before gonadotoxic agents  • IVF setting | • Age-matched male factor infertility  • IVF-ICSI | • Received AI or anti-estrogen therapy  • With autoimmune conditions  • Received earlier chemotherapy or radiotherapy | Basal FSH and AFC | ND |
| Johnson, et al.  (2013) | USA | 2005-2012 | Retrospective cohort | 100 | • Patients with cancer requiring gonadotoxic therapy  • IVF setting | • Age-matched tubal and male factor infertility or oocyte donors  • IVF setting | Previous exposed to chemotherapy | AFC | ND |
| Bala, et al.  (2016) | India | 2013-2014 | Case-control | 60 | BC patients prior to chemotherapy | Age-matched healthy controls | ND | AMH | ND |
| Naasan, et al.  (2016) | Ireland | 2009-2013 | Case-control | 5349 | Patients with cancer prior to chemotherapy, radiotherapy or radical surgery | Control women without cancer experiencing infertility | ND | AMH | Age |
| Paradisi, et al.  (2016b) | Italy | 2011-2015 | Case-control | 82 | • Lymphoma patients without previous cytotoxic treatment  • No ovarian cancer or surgery  • No systemic or endocrine / metabolic  disease  • No recent hormonal therapy | • Age-matched healthy volunteers  • Age: 18 to 39  • Evaluated the same way as the cancer group | ND | AMH | ND |
| Pereira, et al.  (2016) | USA | 2005-2013 | Retrospective  cohort | 671 | • BC patients before the initiation of adjuvant chemotherapy  • Oocyte or embryo cryopreservation | • Age-matched controls without any pertinent medical, surgical or gynecologic history  • Elective oocyte cryopreservation | Previous unilateral oophorectomy or known polycystic ovarian syndrome | AMH | ND |
| Quinn, et al.  (2017) | USA | 2009-2015 | Retrospective cohort | 589 | • BC patients  • No prior history of infertility, and planed treatment with the potential to compromise future fertility  • Oocyte cryopreservation | • Control women with no history of infertility  • Elective oocyte cryopreservation | ND | AFC | Age, BMI and total gonadotropin  dose |
| Decanter, et al.  (2018) | France | 2011-2014 | Prospective cohort | 270 | • Patients with cancer before chemotherapy  • Presence of two ovaries and absence of thrombo-embolic events  • Age ≤ 38  • Discontinuation of OCP  • Oocyte vitrification | • Age-matched healthy controls for male factor  • IVF-ICSI | • Suspected uptake at PET scan  • Breast cancer received neo-adjuvant situation | AMH | ND |
| Goldrat, et al.  (2019) | Belgium | 2012-2017 | Prospective cohort | 47 | • BC patients before chemotherapy  • Age: 18 to 41  • Non-metastatic disease with basal FSH < 20 IU/L  • IVF-ICSI, oocyte/embryo cryopreservation | • Age-matched tubal, male and/or idiopathic  infertility  • IVF-ICSI | • Severe endometriosis  • Ovarian insufficiency  • Severe PCOS  • AMH < 0.5 or > 8 ng/ml | AMH | ND |
| Gunnala, et al.  (2019) | USA | 2010-2015 | Retrospective  cohort | 795 | • Patients with cancer before gonadotoxic therapy  • Oocyte cryopreservation | • Control women without cancer  • Elective egg freezing | • Age > 40 with ovarian cancer  • Prior oophorectom  • Previous gonadooxic therapy  • Chronic medical conditions  • Taking hormone suppression ≥ 3 month | AMH, basal FSH and AFC | Age and BMI |
| Porcu, et al.  (2020) | Italy | 2014-2019 | Prospective cohort | 248 | • Stage I-II BC patients  • Age: 18 to 40  • Performed *BRCA* test  • Oocyte cryopreservation | • Age matched healthy controls for male factor  • Oocyte cryopreservation | • Age < 18 and > 40  • Already had chemotherapy  • BC stage III and IV + / - metastasis  • Cancer patients were nor performed BRCA test | AMH, basal FSH and AFC | Age and BMI |

AUTHOR: edited for journal style.

AMH, anti-Müllerian hormone; FSH, follicle-stimulating hormone; AFC, antral follicle count; hCG, human chorionic gonadotropin; BC, breast cancer; OCP, oral contraception; IVF, in-vitro fertilization; ICSI, intracytoplasmic sperm injection; PCOS, polycystic ovarian syndrome; BMI, body mass index; ND, not disclosed.

| **Supplementary Table S3.** Studies eligible for meta-analysis focusing on the impact of cancer on ovarian reserve test outcome measures | | | | | | | | | | | | |
| --- | --- | --- | --- | --- | --- | --- | --- | --- | --- | --- | --- | --- |
| Author, year | Location | Study design | Cancer type | Number of women | | Mean age (years) | | Mean ORT results | | Unit | Hormonal assay | Diameter for AFC (mm) or calculation for ovarian volume |
|  |  |  |  | Cancer | Control | Cancer | Control | Cancer | Control |  |  |  |
| **AMH** |  |  |  |  |  |  |  |  |  |  |  |  |
| Lutchman Singh et al. (2007) | UK | Cross-sectional | Breast cancer | 9 | 18 | 35.20 ± 1.47^a^ | 34.46 ± 0.87^a^ | 6.77 ± 1.70^a^ | 7.89 ± 1.62^a^ | ng/mL | IOT | / |
| Lie Fong et al.  (2008) | The Netherlands | Case-control | Hematological malignancies | 13 | 42 | 29.40  (16.00 – 36.20)^b^ | 29.90  (19.60 – 35.60)^b^ | 1.00  (0.01 – 2.90)^b^ | 2.10  (0.10 – 7.40)^b^ | μg/L | IOT | / |
| Yu, et al.  (2010) | USA | Prospective  cohort | Breast cancer | 26 | 134 | (30 – 40)^b^ | Age-matched | 0.86  (0.07 – 9.10)^b^ | 0.94  (0.20 – 7.70)^b^ | ng/mL | DSL | / |
| Bala, et al.  (2016) | India | Case-control | Breast cancer | 30 | 30 | Age-matched | Age-matched | 1.67 ± 0.44 | 1.90 ± 0.37 | ng/mL | AMH  Gen II | / |
| Naasan, et al.  (2016) | Ireland | Case-control | Breast cancer | 49 | 5231 | 36.30 ± 5.30 | 35.40 ± 4.40 | 13.18 ± 12.50 | 17.86 ± 21.55 | pmol/L | DSL and AMH  Gen II | / |
|  |  |  | Sarcoma | 5 | 5231 | 34.40 ± 8.00 | 35.40 ± 4.40 | 17.84 ± 5.12 | 17.86 ± 21.55 | pmol/L | DSL and AMH  Gen II | / |
|  |  |  | Endometrial cancer | 2 | 5231 | 35.30 ± 5.00 | 35.40 ± 4.40 | 5.36 ± 3.17 | 17.86 ± 21.55 | pmol/L | DSL and AMH  Gen II | / |
| Paradisi, et al.  (2016a) | Italy | Case-control | Overall cancer^e^ | 191 | 43 | 26.40 ± 6.90 | 28.80 ± 6.20 | 2.80  (1.60 – 4.15)^c^ | 2.80  (1.20 – 4.90)^c^ | ng/mL | AMH  Gen II | / |
| Paradisi, et al.  (2016b) | Italy | Case-control | Hematological malignancies (Lymphoma) | 73 | 25 | 24.30 ± 6.20 | 26.10 ± 5.70 | 2.38 ± 1.85 | 3.34 ± 1.94 | ng/mL | AMH  Gen II | / |
| Pereira, et al.  (2016) | USA | Retrospective  cohort | Breast cancer | 220 | 451 | 36.00  (33.00 – 38.00)^c^ | 37.00  (34.00 – 39.00)^c^ | 1.41  (0.70 - 2.29)^c^ | 1.36  (0.67 - 2.32)^c^ | ng/mL | ND | / |
| Decanter, et al.  (2018) | France | Prospective cohort | Breast cancer | 90 | 180 | 30.8 ± 3.3 | 29.00 ± 5.00 | 34.50 ± 42.20 | 44.00 ± 26.10 | pmol/L | AMH  Gen II | / |
|  |  |  | Breast cancer | 45 | 180 | 30.8 ± 3.3 | 29.00 ± 5.00 | 34.20 ± 37.40 | 44.00 ± 26.10 | pmol/L | AMH  Gen II | / |
| Goldrat, et al.  (2019) | Belgium | Prospective cohort | Breast cancer | 23 | 24 | 30.40 ± 3.80 | 30.80 ± 3.90 | 2.70 ± 2.10 | 3.10 ± 1.70 | ng/dL | ND | / |
| Gunnala, et al.  (2019) | USA | Retrospective  cohort | Breast cancer (BRCA+) | 38 | 19 | 32.70 ± 3.80 | 31.70 ± 3.10 | 2.60 ± 2.10 | 3.20 ± 2.20 | ng/mL | AMH  Gen II | / |
|  |  |  | Overall cancer | 85 | 19 | 29.30 ± 6.10 | 31.70 ± 3.10 | 2.90 ± 3.20 | 3.20 ± 2.20 | ng/mL | AMH  Gen II | / |
| Porcu, et al.  (2020) | Italy | Prospective cohort | Breast cancer (*BRCA1+*) | 11 | 181 | 31.50±3.20 | 32.40 ± 2.80 | 1.20 ± 1.10 | 3.80 ± 2.50 | ng/mL | ND | / |
|  |  |  | Breast cancer (*BRCA2+*) | 11 | 181 | 33.20±4.50 | 32.40 ± 2.80 | 4.40 ± 5.30 | 3.80 ± 2.50 | ng/mL | ND | / |
|  |  |  | Breast cancer (*BRCA-*) | 24 | 181 | 32.50±4.30 | 32.40 ± 2.80 | 4.50 ± 4.10 | 3.80 ± 2.50 | ng/mL | ND | / |
| **Inhibin B** |  |  |  |  |  |  |  |  |  |  |  |  |
| Lutchman Singh et al. (2007) | UK | Cross- sectional | Breast cancer | 9 | 18 | 35.20 ± 1.47^a^ | 34.46 ± 0.87^a^ | 74.97 ± 17.70^a^ | 83.61 ± 13.45^a^ | pg/mL | DSL | / |
| Paradisi, et al.  (2016a) | Italy | Case-control | Overall cancer^e^ | 191 | 43 | 26.4 0± 6.90 | 28.80 ± 6.20 | 41.00  (18.60 – 73.30)^c^ | 43.40  (22.80 – 81.10)^c^ | pg/mL | Inhibin B Gen II | / |
| **Basal FSH** |  |  |  |  |  |  |  |  |  |  |  |  |
| Oktay, et al.  (2006) | USA | Retrospective cohort | Breast cancer | 47 | 56 | 36.40 ± 3.60 | 36.90 ± 3.90 | 7.10 ± 3.10 | 4.20 ± 2.00 | IU/L | ND | / |
| Lutchman Singh et al. (2007) | UK | Cross-sectional | Breast cancer | 14 | 24 | 35.20 ± 1.47^a^ | 34.46 ± 0.87^a^ | 6.09 ± 0.49^a^ | 6.62 ± 0.42^a^ | mIU/mL | DPC | / |
| Quintero, et al.  (2010) | USA | Retrospective  cohort | Overall cancer^e^ | 50 | 50 | 32.30 ± 5.00 | 32.30 ± 5.00 | 7.30 ± 2.50 | 6.30 ± 2.10 | IU/L | ND | / |
|  |  |  | Breast cancer | 28 | 50 | 34.00 ±3.20 | 32.30 ± 5.00 | 7.40 ± 2.50 | 6.30 ± 2.10 | IU/L | ND | / |
| Das, et al.  (2011) | Canada | Retrospective cohort | Hematological malignancies | 19 | 48 | 27.50 ± 1.20^a^ | 30.70 ± 0.30^a^ | 6.20 ± 0.50^a^ | 6.80 ± 0.30^a^ | IU/L | ND | / |
|  |  |  | Brain cancer | 5 | 48 | 29.40 ± 0.80^a^ | 30.70 ± 0.30^a^ | 7.40 ± 0.50^a^ | 6.80 ± 0.30^a^ | IU/L | ND | / |
|  |  |  | Bone cancer | 5 | 48 | 29.50 ± 2.80^a^ | 30.70 ± 0.30^a^ | 6.90 ± 1.30^a^ | 6.80 ± 0.30^a^ | IU/L | ND | / |
| Gunnala, et al.  (2019) | USA | Retrospective  cohort | Breast cancer (BRCA+) | 38 | 19 | 32.70 ± 3.80 | 31.70 ± 3.10 | 6.70 ± 2.40 | 6.40 ± 2.70 | mIU/mL | ND | / |
|  |  |  | Overall cancer | 85 | 19 | 29.30 ± 6.10 | 31.70 ± 3.10 | 4.80 ± 2.80 | 6.40 ± 2.70 | mIU/mL | ND | / |
| Porcu, et al.  (2020) | Italy | Prospective cohort | Breast cancer (*BRCA1+*) | 11 | 181 | 31.50 ± 3.20 | 32.40 ± 2.80 | 6.20 ± 3.80 | 7.10 ± 2.20 | mIU/mL | ND | / |
|  |  |  | Breast cancer (*BRCA2+*) | 11 | 181 | 33.20 ± 4.50 | 32.40 ± 2.80 | 5.90 ± 1.90 | 7.10 ± 2.20 | mIU/mL | ND | / |
|  |  |  | Breast cancer (*BRCA-*) | 24 | 181 | 32.50 ± 4.30 | 32.40 ± 2.80 | 6.50 ± 2.70 | 7.10 ± 2.20 | mIU/mL | ND | / |
| **AFC** |  |  |  |  |  |  |  |  |  |  |  |  |
| Lutchman Singh et al. (2007) | UK | Cross-sectional | Breast cancer | 13 | 20 | 35.20 ± 1.47^a^ | 34.46 ± 0.87^a^ | 16.77 ± 1.63^a^ | 17.05 ± 1.21^a^ | / | / | 2-10mm total |
| Das, et al.  (2011) | Canada | Retrospective cohort | Hematological malignancies | 19 | 48 | 27.50 ± 1.20^a^ | 30.70 ± 0.30^a^ | 18.60 ± 2.40^a^ | 17.40 ± 1.40^a^ | / | / | ND |
|  |  |  | Brain cancer | 5 | 48 | 29.40 ± 0.80^a^ | 30.70±0.30^a^ | 19.80 ± 1.90^a^ | 17.40 ± 1.40^a^ | / | / | ND |
|  |  |  | Bone cancer | 5 | 48 | 29.50±2.80^a^ | 30.70±0.30^a^ | 21.80 ± 6.40^a^ | 17.40 ± 1.40^a^ | / | / | ND |
| Johnson, et al.  (2013) | USA | Retrospective cohort | Overall cancer^e^ | 22 | 22 | ND | ND | 21.90  (17.10 – 26.60)^d^ | 21.10  (17.20 – 25.10)^d^ | / | / | ND |
| Paradisi, et al.  (2016a) | Italy | Case-control | Overall cancer^e^ | 191 | 43 | 26.40 ± 6.90 | 28.80 ± 6.20 | 15.0  (10.00 – 20.00)^c^ | 17.00  (9.00 – 21.00)^c^ | / | / | 2-9mm total |
| Quinn, et al.  (2017) | USA | Retrospective cohort | Breast cancer  (18-34y) | 83 | 81 | (18 – 34)^b^ | (18 – 34)^b^ | 19.10 ± 11.80 | 18.30 ± 13.50 | / | / | ND |
|  |  |  | Breast cancer  (35-37y) | 42 | 188 | (35 – 37)^b^ | (35 – 37)^b^ | 14.70 ± 8.20 | 16.00 ± 9.50 | / | / | ND |
|  |  |  | Breast cancer  (38-40y) | 45 | 101 | (38 – 40)^b^ | (38 – 40)^b^ | 11.40 ± 8.10 | 13.30 ± 7.40 | / | / | ND |
|  |  |  | Breast cancer  (40-42y) | 18 | 23 | (40 – 42)^b^ | (40 – 42)^b^ | 10.00 ± 7.40 | 11.10 ± 6.60 | / | / | ND |
| Decanter, et al.  (2018) | France | Prospective cohort | Overall cancer^e^ | 90 | 180 | 30.8 ± 3.3 | 29.00 ± 5.00 | 23.90 ± 17.50 | 33.60 ± 16.60 | / | / | 2-9mm total |
| Gunnala, et al.  (2019) | USA | Retrospective  cohort | Breast cancer (BRCA+) | 38 | 19 | 32.70 ± 3.80 | 31.70 ± 3.10 | 15.20 ± 5.00 | 16.30 ± 3.90 | / | / | ND |
|  |  |  | Overall cancer | 85 | 19 | 29.30 ± 6.10 | 31.70 ± 3.10 | 14.70 ± 6.80 | 16.30 ± 3.90 | / | / | ND |
| Porcu, et al.  (2020) | Italy | Prospective cohort | Breast cancer (*BRCA1+*) | 11 | 181 | 31.50 ± 3.20 | 32.40 ± 2.80 | 11.80 ± 8.50 | 12.48 ± 1.40 | / | / | ND |
|  |  |  | Breast cancer (*BRCA2+*) | 11 | 181 | 33.20 ± 4.50 | 32.40 ± 2.80 | 11.50 ± 4.90 | 12.48 ± 1.40 | / | / | ND |
|  |  |  | Breast cancer (*BRCA-*) | 24 | 181 | 32.50 ± 4.30 | 32.40 ± 2.80 | 14.20 ± 7.50 | 12.48 ± 1.40 | / | / | ND |

All values are expressed as means ± standard deviation (SD) except for the additional markers. AMH, anti-Müllerian hormone; FSH, follicle-stimulating hormone; AFC, antral follicle count; IOT, Immunotech; DSL, Diagnostic Systems Laboratories; DPC, diagnostic products company; CI, confidence interval; ND, not disclosed.

^a^Values are mean ± standard error of the mean (SEM).

^b^Values are median (range)

^c^Values are median (interquartile range, IQR)

^d^Values are mean (95% CI)

^e^Overall cancer is defined when cancer type is not described in detail.

^f^D1, D2 and D3 are maximal perpendicular diameters of the ovary.

| **Supplementary Table S4.** Standard or weighted mean difference and 95% CI for the association between cancer and ovarian reserve test biomarkers | | | | | |
| --- | --- | --- | --- | --- | --- |
|  | Number of studies | SMD or WMD (95% CI) | *I^2^* (%) | *P^a^* | *P^b^* between |
| **AMH**^c^ |  |  |  |  |  |
| All studies | 17 | -0.19 (-0.34, -0.03) | 53.8 | 0.004 |  |
| By studies design |  |  |  |  |  |
| Case-control | 7 | -0.32 (-0.51, -0.12) | 17.1 | 0.300 | 0.223 |
| Cross-sectional | 1 | -0.18 (-0.98, 0.63) | - | - |  |
| Cohort | 9 | -0.09 (-0.31, 0.13) | 63.8 | 0.005 |  |
| Retrospective cohort | 3 | -0.01 (-0.16, 0.13) | 0.0 | 0.0564 | 0.254 |
| Prospective cohort | 6 | -0.10 (-0.47, 0.27) | 75.3 | 0.001 |  |
| By geographic location |  |  |  |  |  |
| North America | 4 | 0.03 (-0.17, 0.24) | 26.0 | 0.006 | 0.729 |
| Europe | 12 | -0.25 (-0.44, -0.05) | 46.3 | 0.039 |  |
| Asia | 1 | -0.57 (-1.08, -0.05) | - | - |  |
| By AMH assay |  |  |  |  |  |
| IOT | 2 | -0.54 (-1.16, 0.08) | 32.8 | 0.222 | 0.500 |
| DSL | 1 | 0.37 (-0.05, 0.80) | - | - |  |
| AMH Gen II | 6 | -0.27 (-0.43, -0.11) | 0.0 | 0.502 |  |
| DSL and AMH Gen II | 3 | -0.21 (-0.47, 0.05) | 0.0 | 0.781 |  |
| Not report | 5 | -0.11 (-0.47, 0.25) | 70.9 | 0.008 |  |
| ROBINS-I risk of bias |  |  |  |  |  |
| Low/moderate | 9 | -0.19 (-0.34, -0.04) | 28.3 | 0.193 | 0.656 |
| Serious/critical | 8 | -0.17 (-0.51, 0.17) | 69.9 | 0.002 |  |
| By cancer type |  |  |  |  |  |
| Overall cancer^e^ | 2 | -0.20 (-0.42, 0.02) | 15.7 | 0.276 | 0.903 |
| Breast cancer | 10 | -0.13 (-0.35, 0.10) | 62.2 | 0.005 |  |
| Hematological malignancies | 2 | -0.62 (-0.99, -0.24) | 0.0 | 0.452 |  |
| Sacorma | 1 | -0.00 (-0.88, 0.88) | - | - |  |
| Endometrial carcinoma | 1 | -0.58 (-1.97, 0.81) | - | - |  |
| Non-breast-cancer malignancies | 1 | -0.10 (-0.60, 0.40) | - | - |  |
| **Basal FSH**^d^ |  |  |  |  |  |
| All studies | 11 | 0.00 (-0.85, 0.85) | 79.3 | < 0.001 |  |
| By studies design |  |  |  |  |  |
| Case-control | 3 | 0.01 (-0.80, 0.81) | 5.6 | 0.347 | 0.916 |
| Cross-sectional | 1 | -0.53 (-1.79, 0.73) | - | - |  |
| Cohort | 7 | 0.05 (-1.22, 1.31) | 86.4 | < 0.001 |  |
| Retrospective cohort | 4 | 0.69 (-1.07, 2.46) | 89.4 | <0.001 | 0.140 |
| Prospective cohort | 3 | -0.89 (-1.65, -0.13) | 0.0 | 0.769 |  |
| By geographic location |  |  |  |  |  |
| North America | 7 | 0.44 (-0.69, 1.57) | 82.6 | < 0.001 | 0.149 |
| Europe | 4 | -0.79 (-1.45, -0.14) | 0.0 | 0.860 |  |
| Asia | 0 | - | - | - |  |
| ROBINS-I risk of bias |  |  |  |  |  |
| Low/moderate | 4 | -0.14 (-0.80, 0.52) | 0.0 | 0.453 | 0.829 |
| Serious/critical | 7 | 0.05 (-1.22, 1.31) | 86.4 | < 0.001 |  |
| By cancer types |  |  |  |  |  |
| Overall cancer^e^ | 2 | -0.25 (-0.28, 2.30) | 89.8 | 0.002 | 0.945 |
| Breast cancer | 6 | 0.05 (-1.38, 1.47) | 86.1 | < 0.001 |  |
| Hematological malignancies | 1 | -0.60 (-1.74, 0.54) | - | - |  |
| Brain cancer | 1 | 0.60 (-0.54, 1.74) | - | - |  |
| Bone cancer | 1 | 0.10 (-2.52, 2.72) | - | - |  |

The proportion of the heterogeneity attributed to between-study variation rather than owing to chance is assessed by I^2^ (%), and I^2^ with the values of 25%, 50% and 75% provides evidence for low, moderate and high between-heterogeneity, respectively. CI, confidence interval; AMH, anti-Müllerian hormone; FSH, follicle-stimulating hormone.

^a^P-value explaining for heterogeneity among the studies within ovarian function biomarker.

^b^P-value testing for Anticipating heterogeneity between subgroup or category investigated by meta-regression analysis.

^c^All analyses were based on SMD.

^d^All analyses were based on WMD.

^e^Overall cancer is defined when cancer type is not described in detail.
